# Development and effectiveness evaluation of an interactive e-learning environment to enhance digital health literacy in cancer patients: study protocol for a randomized controlled trial

**DOI:** 10.3389/fdgth.2025.1455143

**Published:** 2025-01-24

**Authors:** Lukas Lange-Drenth, Hellena Willemer, Mirjam Banse, Anke Ernst, Anne Daubmann, Anja Holz, Christiane Bleich, Susanne Weg-Remers, Holger Schulz

**Affiliations:** ^1^ Department of Medical Psychology, University Medical Center Hamburg-Eppendorf, Hamburg, Germany; ^2^ Division Cancer Information Service, German Cancer Research Center (DKFZ), Heidelberg, Germany; ^3^ Institute of Medical Biometry and Epidemiology, University Medical Center Hamburg-Eppendorf, Hamburg, Germany

**Keywords:** digital health, eHealth, health literacy, eHealth literacy, health information, e-learning, medical education, digital health literacy

## Abstract

**Background:**

The Internet allows cancer patients to access information about their disease at any time. However, the quality of online information varies widely and is often inaccurate or does not provide all the details patients need to make informed decisions. Additionally, patients’ often limited ability to find and evaluate cancer-related online information can lead to misinformation.

**Objective:**

An interactive e-learning environment to promote digital health literacy will be developed and evaluated for effectiveness.

**Primary hypothesis:**

Cancer patients who use the e-learning environment (IG1.1–IG1.3) or the content of the environment as a non-interactive PDF file (IG2) will show greater improvement in their digital health literacy from baseline to 8 weeks after baseline compared to patients who receive no such intervention, but are referred to a standard information brochure.

**Methods:**

The hypothesis will be tested in a stratified randomized controlled superiority trial with five parallel groups and the primary endpoint of digital health literacy. In an e-learning environment, patients will learn strategies to use when searching for reliable cancer-related online information. During development, a prototype will be refined through focus groups and tested for usability by experts and patients. 660 cancer patients will be recruited using convenience sampling and randomly assigned in a 3:1:1 ratio to IG1.1-IG1.3 (three variants of the environment), IG2, or the control group. Two thirds of the 660 participants will be recruited through the German Cancer Information Service (CIS) and one third through non-CIS routes. Allocation will follow stratified randomization, accounting for recruitment route (CIS vs. non-CIS) and cancer type (breast cancer vs. other cancers), with variable block length. The primary outcome, digital health literacy, will be measured at baseline, 2 weeks, and 8 weeks after baseline.

**Conclusion:**

If the results support the primary hypothesis, then the e-learning environment could empower patients to retrieve more reliable information about their disease. Concerns about the generalizability of the results, since a disproportionate number of inquiries to the CIS come from breast cancer patients, are addressed by a proportionally stratified randomization strategy and diversified recruitment routes.

## 1 Introduction

### 1.1 Background

The need for information is one of the most common unmet support needs of cancer patients during the course of the disease (1). Most cancer patients want to receive all available information about their disease and treatment (2, 3) and the proportion of cancer patients using the internet to search for cancer-related information is high (>70%) (4–6) and will continue to increase in the future (7). In Germany in 2019, even in the 65+ age group, 68% had searched for health-related information on the internet in the previous 3 months (8). Approximately 20% of cancer patients use health-related discussion forums or blogs (6) and about 35% reported using social media (6, 9).

Searching for health-related information on the internet is positively associated with patient-reported outcome measures (PROMs) and socioeconomic characteristics of patients. Cross-sectional studies indicate that cancer patients who search the internet for cancer-related information are more involved in medical decision-making (10, 11), feel better informed about their disease (5), have higher levels of self-reported health (12) and quality of life (QoL) (13), are more likely to have a partner (6), are younger and more educated (4, 6, 13) than patients who do not search the internet. Depending on whether patients discuss health-related online information with their physician, searching for health information online can also improve the doctor-patient relationship for patients with acute or chronic conditions (14).

The quality of cancer-related information available on information websites, social media, and from artificial intelligence (AI) chatbots varies. Information on websites, as measured by the DISCERN instrument developed to help consumers evaluate the quality of health-related information about treatment options for a health problem, is often interest-driven, incomplete, or out-of-date. Additionally, such information may not provide all the details necessary for cancer patients to properly assess its relevance in the context of their individual disease and make informed decisions with their healthcare provider based on the information received (15–18). More research is needed on the quality of information posted on social networking sites such as Facebook or Instagram and on discussion forums and blogs, where informal advice and support is given by layman without quality control. Low to moderate quality and high rates of misinformation for prostate and bladder cancer measured by the DISCERN instrument have been found on several social networks, including TikTok, Instagram, and YouTube (19, 20). Encouragingly, studies results show that the dissemination of evidence-based information and social support through X-messaging is associated with an increase in self-perceived cancer-related knowledge (21), that participation in social media support groups can improve breast cancer patients’ understanding of their disease and its management (21), and that frequently shared (retweeted) X-messages are more likely to contain medically accurate information than randomly selected X-messages (22). Initial studies concerning AI chatbots suggest that for the top cancer-related searches for prostate, bladder, kidney, and testicular cancer, AI chatbots produce information that may be partially accurate and of moderately high quality as measured by the DISCERN instrument. However, their responses are quite difficult to read, moderately difficult to understand, and lack clear instructions for users to follow (23, 24). Deviating or even incorrect information, for example on treatment recommendations, were found in a certain percentage of responses, using the National Comprehensive Cancer Network (NCCN) (25) guidelines as a benchmark (26). Assessment tools for health-related (online) information that patients can use freely, such as the DISCERN criteria, have shortcomings (1): low interrater reliability (27), (2) subscales with only one item, i.e., with only limited reliability (28) and (3) the ratings are highly subjective (28).

Due to the varying quality of health-related online information, patients therefore depend on their own ability to find and critically evaluate online cancer information. These skills are part of the concept of eHealth literacy or digital health literacy. A systematic review shows that eHealth literacy levels among older adults are positive associated with behavioral and cognitive outcomes, such as health-promoting behaviors, decision-making, and health knowledge. However, evidence for physical and psychological outcomes is inconsistent, highlighting the need for high-quality studies to better understand these relationships (29). The overall eHealth literacy level among cancer survivors is not high, and influenced by factors such as age, gender, education and social support (30). An early definition of eHealth literacy described it as “the ability to seek, find, understand, and appraise health information from electronic sources and apply the knowledge gained to addressing or solving a health problem” (31). This definition served as the foundation for developing the eHealth Literacy Scale (eHEALS) (32), which has become the most widely used and translated questionnaire for assessing eHealth literacy (33). However, although the authors of the eHEALS conceptualized eHealth literacy as encompassing six core skills, the scale itself is unidimensional, which does not align with their multidimensional framework. Performance test analyses of the digital health literacy of patients with cancer and rheumatoid arthritis identified five types of skills that are essential for successfully searching for cancer-related information on websites (34, 35): (1) operational skills, to use the digital device and Internet browser, (2) navigation skills, to navigate back and forth between websites and orientate on the world wide web, (3) information searching skills, to use correct search strategies, i.e., to formulate appropriate search terms, (4) evaluating reliability of online information, i.e., review the source of information (5) evaluating relevance of online information (34, 35). In addition, for interactive technologies, such as forums, e-consults, patient portals, or digital treatment applications, patients need the two skills of (6) adding self-generated content to the Internet and (7) considering their own and others’ privacy, i.e., knowing who can read what they have posted on the Internet (35). These seven skills form the foundation of digital health literacy measured with the Digital Health Literacy Instrument (DHLI) (35), a validated tool designed to measure digital health literacy comprehensively. The DHLI has been translated into multiple languages and adapted for various populations, ensuring its applicability across diverse settings (33). Results of the performance based studies show that the level of information and evaluation skills was significantly lower than the level of operating and navigation skills (34, 36). Many participants had problems with formulating task-related search terms (90%) and selecting a task-related search result (52%) (34). These findings are consistent with the results of a survey in a representative sample of the German population (37) where three quarters (76%) of respondents had low digital health literacy (37). Potential confounders of the digital health literacy or eHealth literacy in healthy individuals and cancer patients are age, education and self-reported health status. Higher digital health literacy or eHealth literacy can be found in young people with higher education (30, 34–38), and higher self-reported health (35). Limitations of self-reported questionnaires, such as the DHLI and eHEALS, might also be that self-reported skills are sometimes only weakly associated with the ability to perform eHealth tasks effectively (39). Additionally, study results suggest that individuals tend to overestimate their computer skills (40, 41) as well as their ability to locate and evaluate web-based information (42).

In summary, cancer patients are a vulnerable group who need support in finding and evaluating online information due to their high information needs, the variable quality of cancer-related online information, and the fact that the majority do not have the necessary information search and credibility assessment skills to handle cancer-related information online correctly. An e-learning environment to improve online health literacy is an effective way to reach as many patients as possible and participants can learn and gain information easily from anywhere. We interpret e-learning in its broadest sense as “instructions delivered on a digital device that is intended to support learning” (43). This encompasses a diverse array of digital training tools, including web-based learning modules and simulation training conducted in virtual environments. A key strength of e-learning is its support for autonomous learning, enabling individuals to set goals, choose resources, and self-assess progress. It also allows for repeated review of materials and seamless content updates to maintain relevance and accuracy (44, 45). While e-learning is commonly used for knowledge transfer, it also serves as an effective platform for skills-based training (46). An initial study using an e-learning environment to improve participants’ digital health literacy indicates short-term effectiveness of the program. 134 healthy Japanese participated in interactive e-learning environments (47), which included information on: (1) reliability of information on the Internet, (2) scientific research methods, and (3) cautions regarding health information posted on social networking websites. After 2 weeks participants in the intervention group improved their self-perceived digital health literacy significantly more (Cohen's *d* = 0.25) than participants in the control group who received no intervention (47).

Interactive and persuasive elements to increase participants’ motivation and engagement are important components in an interactive e-learning environment designed to increase participants’ digital health literacy. Research suggests that online courses with high levels of interactivity lead to higher levels of students’ motivation, improved learning outcomes, and satisfaction than less interactive learning environments (48–51). Simple digital interactions, such as choosing when to start each segment of the presentation and answering questions about the content, have shown to positively impact learning outcomes, motivation and satisfaction with the course compared to passive learning approaches (50–52). Motivation and engagement are factors that are correlated and to some extent overlapping. Motivation can mediate the success of an e-learning intervention (53). High levels of intrinsic motivation for e-learning environments are associated with higher levels of engagement (54). Higher levels of engagement are in turn associated with higher intervention effectiveness (55). Engagement is a complex construct composed of behavioral (e.g., system usage data), cognitive (e.g., intervention credibility and motivation), and affective (e.g., satisfaction with the intervention, acceptability, and feasibility) dimensions and is therefore difficult to measure (56).

One way to improve user motivation and engagement is the use of various persuasive elements (57, 58). Persuasive systems, designed to influence users’ attitudes or behaviors, follow a structured design process, such as in the Persuasive Systems Design (PSD) model (59). This model suggests the implementation of various persuasive elements in four categories of support. In modern e-learning environments, the following persuasive elements may be used (1): the primary task support of e- learning environments is to support the user in acquiring new knowledge. Possible elements of primary task support are: (a) “reduction”: reducing complex behaviors to simple tasks; (b) “rehearsal”: providing the means to practice or repeat a behavior; and (c) “tunneling”: guiding users through a process or experience (58). (2) The credibility of e-learning environments is largely based on the user's trust in the information provider. Health-related websites operated by university institutions generally enjoy a high level of trust among users (60). Possible elements in the credibility support of e-learning environments are (a) “authority”: responsible persons should be named in the system; and (b) “verifiability” content in the system should be easily verifiable through listed external sources (58). (3) Dialog support and social support only take place to a limited extent in e-learning environments. Possible elements are: (a) “Praise”: giving positive feedback via words, images, symbols or sounds; and (b) “Rewards”: reward for performing a target behavior (58). A systematic review found a correlation between the number of persuasive elements and the effectiveness of web-based interventions (61). However, this finding should not be interpreted to mean that implementing more principles always leads to better outcomes. Rather, persuasive elements should be tested with potential end-users in the pilot phase.

E-learning environments are part of the overarching concept of eHealth (62). Although there is growing evidence in systematic reviews of the positive impact of eHealth interventions on the health of patients with chronic conditions (63, 64) the actual implementation of such programs in everyday clinical practice has proven to be a challenge (65). The distinction between the effectiveness of the implementation and the basic experimental effectiveness of the intervention (efficacy) is of crucial importance for the transfer of the interventions from the study situation to real-life care (66). Proctor (66) proposed eight dimensions to evaluate the implementation of interventions in health care: Acceptability, Adoption, Appropriateness, Feasibility, Fidelity, Cost, Penetration, and Sustainability. The implementation of interventions should be evaluated against these eight dimensions in order to obtain accurate information about the implementation process and identify potential barriers.

In summary, cancer patients often lack the digital health literacy needed to effectively find, evaluate, and use online health information. Despite their high information needs, there are no targeted interventions for this group, even as national and international health strategies prioritize improving health literacy. Addressing this gap through tailored e-learning interventions could significantly enhance cancer patients’ health outcomes and quality of life.

### 1.2 Objectives

We will investigate the following primary research question: (1) can cancer patients improve their digital health literacy from baseline (T0) to 8 weeks after baseline (T2) by using an interactive e-learning environment (IG1.1–IG1.3) or a non-interactive PDF file (IG2) that provides the same content as the interactive learning environment, compared to a control group? In addition, we will investigate whether (2) the primary question also applies to the course from baseline to 2 weeks after baseline (T1); (3) whether the primary question also applies with the total score of the performance-based items of the DHLI as the dependent variable; (4) whether and - if so - which elements of the primary task support, i.e., reduction and tunneling, influence the effectiveness of the intervention.; (5) whether participants’ self-reported motivation mediates the relationship between the intervention group and the change in digital health literacy from T0 to T2; (6) whether cancer patients perceive the interactive, digital e-learning environment as user-friendly, appropriate and feasible, and (7) whether cancer patients perceive the e-learning environment as more user-friendly, appropriate and feasible than the non-interactive PDF file.

#### 1.2.1 Hypotheses

The primary hypothesis is: Cancer patients who use a digital interactive e-learning environment (IG1.1–IG1.3) or the content of the e-learning environment as a non-interactive PDF file (IG2) will show a greater improvement in their digital health literacy from T0 to T2 as compared to cancer patients who receive no intervention. The secondary hypothesis is: cancer patients who use the full e-learning environment (IG1.1) will show a greater improvement in their digital health literacy from T0 to T2 compared to cancer patients who use the e-learning environment without tunneling elements (IG1.2) or the e-learning environment without rehearsal elements (IG1.3).

## 2 Methods

### 2.1 Study design

**Figure 1:**
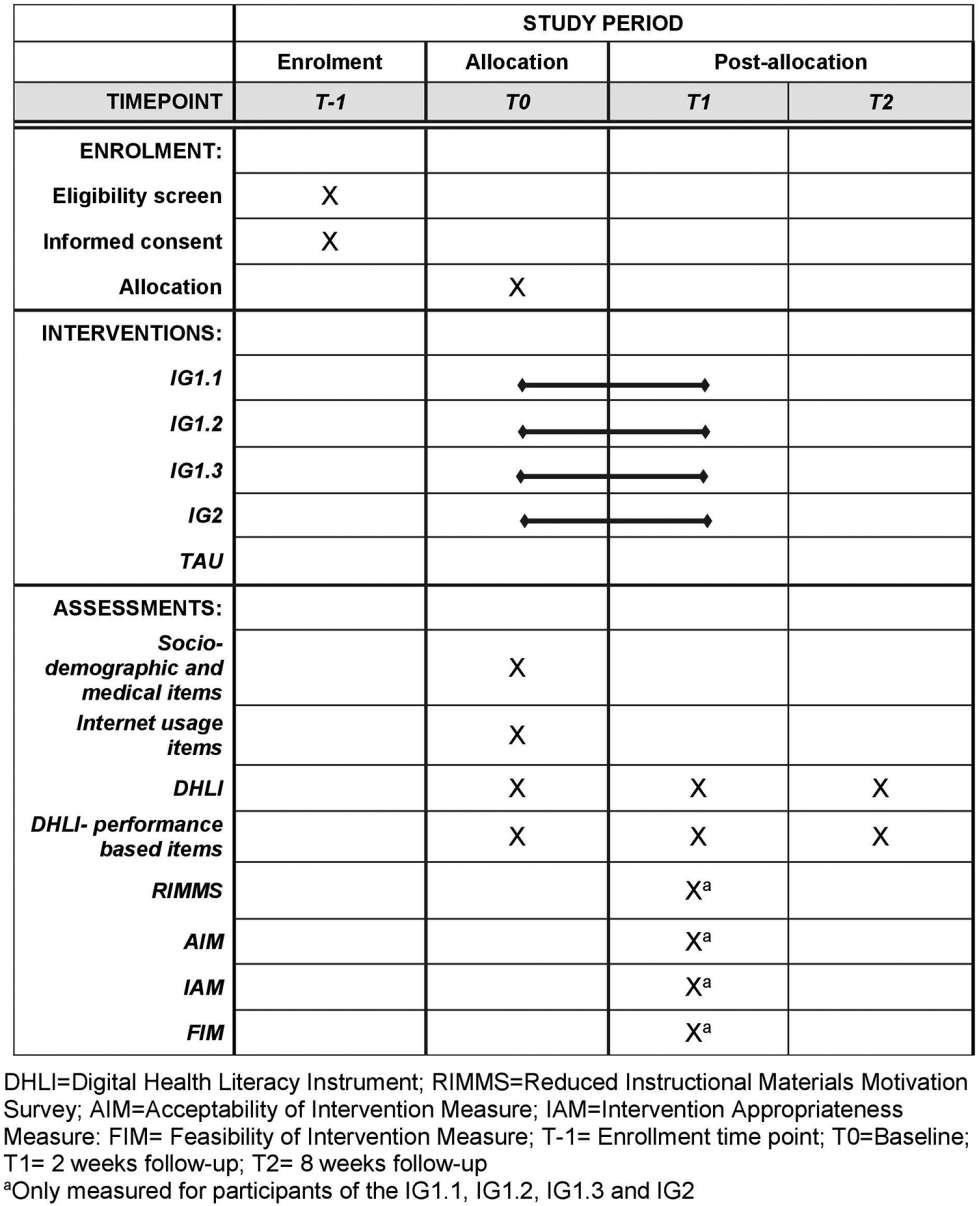
Participant timeline (spirit figure) of enrolment, interventions, and assessments.

This trial is designed as a stratified randomized controlled superiority trial with five parallel groups and the primary endpoint of digital health literacy. Participants in intervention groups one, two, and three (IG1.1–IG1.3) will receive access to an e-learning environment. Participants in intervention group four (IG2) will receive the content in a non-interactive PDF format. Participants in the control group will not receive any intervention but will be provided with a standard informational brochure. The study also involves the development and pre-testing of the intervention. During development, two focus groups will explore cancer patients’ preferences for intervention topics and design elements to improve digital health literacy. A prototype e-learning environment will undergo usability testing with patient and expert cognitive walkthroughs. The randomization will be performed as a proportionally stratified randomization with variable block length and a 1:1:1:1:1 allocation. The randomization sequence is computer-generated and securely stored within the REDCap system, which ensures that neither participants nor recruiting researchers have access to it. Both, participants and those assessing the outcomes, are unaware of the treatment allocation (blinding). PROMS will be administered at T0, T1 and T2 (Figure 1). The reporting of this study protocol followed the recommendations of the Standard Protocol Items: Recommendations for Interventional Trials (SPIRIT) (67) (Supplementary Table 1).

### 2.2 Eligibility criteria

Patients are eligible for the trial if they are:

- 18 years of age or older
- self-report having been diagnosed with cancer of any type
- have sufficient knowledge of the German, as all study content and questionnaires will be in German only
- should be able to use a digital device (smartphone, tablet, PC, laptop, etc.) with an Internet connection, as all study content and questionnaires will only be accessible online
- must have their own email address, as all reminders for follow-up questionnaires will be sent via email.

Patients will be excluded, if they are:

- severely cognitively impaired due to their cancer or other illnesses and/or are unable to operate a digital device cannot participate in the study.

### 2.3 Setting and recruitment

The enrollment period for the main study will extend over 6 months. Two thirds of the planned 660 participants will be recruited through online recruitment routes of the German Cancer Information Service (CIS), such as email, the CIS website (7.7 million visitors per year), the CIS Facebook page or the CIS Instagram page. One third of the participants will be recruited primarily in Hamburg through in a physical setting through recruitment routes not associated with the CIS, such as oncological outpatient clinics of the University Cancer Center Hamburg (UCCH) or patient support groups. Recruitment via the CIS is organized in cooperation between researchers at the University Medical Center Hamburg-Eppendorf (UKE) and the CIS's Internet Editorial Team. Patients who email the CIS for cancer-related advice will receive an invitation to participate in the study and a link to the contact website (https://www.uke.de/orientiertinformiert) in the reply from the CIS, where patients can enter their contact information (last name, first name, and email address). The contact website provides an overview of the study procedure, outlines the study's goals, specifies the inclusion and exclusion criteria, and allows patients to access the contact information of the researchers. An invitation to participate in the study and a link to the contact website will be posted on the CIS website and on the CIS Facebook and Instagram pages (currently approximately 5,800 and 4,300 followers, respectively). Patients in the waiting room of the UCCH outpatient clinic will be approached by one researcher of the study team who will hand them a card resembling a business card. Patients will be addressed without prior identification, acknowledging the possibility that the individual may not be a patient. This card will include a QR code and URL that patients can use to access the study's contact website. Additionally, the researcher will give the patients a brief description of the study, explain its goals, and address any questions they might have. An invitation to participate in the study and the link to the contact website will also be emailed to support group representatives, who will forward the email to their members. Broad recruitment through the various access channels is used to minimize sample bias by attempting to include patients of lower socioeconomic status, and thus potentially lower digital health literacy, in the study. No incentives will be used to recruit participants. Contact information provided on the contact page will be sent to the project email address. Patients will receive individualized access to the electronic data capture software REDCap (68) via the project email address.

In REDCap, patients will be given access to the informed consent form and, after signing, to the baseline questionnaire. In the consent form, patients will receive the following information about participation in the study and the processing of their personal data: (1) Participants are informed that participation in the study is voluntary and that consent to participate can be withdrawn at any time prior to anonymization without giving reasons; (2) the aim and the purpose of the study are communicated; (3) the procedure and scope of the study (time required) (4) which personal data (age, gender, education, type of cancer, self-assessed health) will be collected (5) the person responsible for data processing and their contact details will be specified; (6) the rights of participants and (7) the date for deletion of personal data (10 years after publication).

### 2.4 Setting and recruitment for pilot studies

The process of recruiting participants, including patients for focus groups, cognitive walkthroughs of the e-learning environment, and pre-testing of questionnaires, will involve collaboration with patient support group representatives, Instagram stories from the CIS and Facebook posts from the CIS. Invitations to participate in the study, along with a link to the contact website, will be emailed to patient support group representatives, who will then email them to their members. CIS will post Instagram stories and Facebook posts with the invitation to participate in the study and a web link to the contact website. Patients interested in participating will provide their contact information (including last name, first name, and email address) through the contact website. The research assistant will schedule a meeting with the patients via E-Mail to conduct the pretests. Patients will be asked to sign an informed consent form before the pre-test begins. In the first focus group, only cancer patients with low digital health literacy will participate. Two items of the HLS-GER 2 (item 1: “Evaluate how trustworthy the information you found is”; item 22: “Use the right words or search terms to find the information you need online”) (37) will be used. Patients will participate in the first focus group if they answer one of the two questions with “very difficult” (about 30% of the population) (37). By including patients with low digital health literacy in the early design process, we hope to increase the uptake of the intervention in this group. The participants in the second focus group and the patient-based cognitive walkthrough should differ on as many relevant factors as possible, especially on digital health literacy (based on the two HLS-GER 2 items) as well as on potential confounders (age and education) of digital health literacy (purposive sampling).

### 2.5 Intervention

#### 2.5.1 Intervention content

The final e-learning environment will include text, audio instruction (with the text read aloud to support participants with varying literacy levels), images, and short videos. These materials were created using Articulate Rise 360, an easy-to-use e-learning authoring tool. The content of the e-learning environment was based on extensive research and guidelines (34, 69–77), designed to improve the different skills of digital health literacy:

(1) Evaluating reliability skills: Participants will get to know criteria and indicators for evaluating the content of the information they find and for assessing the trustworthiness of website providers. This includes, for example, to check for compliance with the transparency criteria for “good health information” (e.g., identification of the operator of the website and the cooperation partners, funding, and thus potential conflicts of interest, qualifications of authors, methodology of information production, and sources on which the information is based) (78). Moreover, participants are taught the following indicators for checking the quality of content and trustworthiness of providers, among others: Checking information against other websites. Checking whether evidence-based data sources are mentioned and whether the information is up-to-date and how often is it updated. Balance and completeness of the information provided, i.e., are the modes of action, benefits and risks of treatment procedures, and consequences of non-treatment described? What is the purpose of the website, who is the target group (79, 80)? Participants will be informed about warning signs of unreliable content: simplified messages; emotional and/or fear-mongering, unbalanced information, extreme adjectives; the report/article relies heavily on subjective information or personal experience.

Information in social media can be evaluated using the following criteria: Who follows the account? Are there contact details? Is there a reference to respectful interaction? Are advertising and editorial content clearly separated? Are there references to the topic, goal or target group? Evaluate content: Can the accuracy of the information be confirmed by other sources? Can you find similar content elsewhere? If so, is evidence-based data mentioned? (81).

(2) Improving information skills: Participants will learn simple rules on how to optimize their Google searches: use more keywords since research shows that cancer patients often use only single-word searches (34); use search operators such as OR; delete irrelevant search terms; introduction to advanced search. They will also learn simple rules to better identify which pages might be relevant in Google search results and which are not: how to recognize Google ads; view URL; view Google “snippet”.
(3) Improving navigation skills: Participants will learn simple rules for navigation; using the back button; using more than one tab; drop-down lists and anchor links are introduced. The environment will cover tips for navigating on different devices like computers, tablets, and smartphones, as these can work differently.
(4) Additional goals are to raise awareness about various aspects related to online searches, including the exploration of alternative and complementary therapies, and the importance of data protection. Finally, a summarizing checklist with the most important criteria to take into consideration when searching for reliable information and links to reliable websites are provided.

Several persuasive and interactive elements will be integrated into the learning environment to increase user motivation and engagement (57, 59). The following primary task support elements are planned for the e-learning environment (82, 83): (1) The complex task of searching for cancer-related information online will be broken down into smaller steps (e.g., how do I check if the information is up-to-date?) (reduction). (2) The participant is guided step-by-step through the separate chapters of the educational content (tunneling) and (3) then repeated or tested in performance tests or quizzes (rehearsal).

The responsible organization and the developers will be named at various points within the learning platform, which should improve the credibility of the learning platform (authority). At the same time, sources of information are provided within the application that enable participants to verify the content they have learned or to acquire additional knowledge (verifiability). Users will also receive praise in words after completing each chapter (praise).

There will be three different versions of the e-learning environment, tailored to examine different instructional features. Participants in the IG1.1 will receive the e-learning environment with all primary task support elements described. Participants in IG1.2 will receive the same content but will not be guided through the content step by step in a predetermined sequence (tunneling), but can determine the order of the content themselves. For instance, participants could skip content or navigate the e-learning material in reverse order, promoting self-directed learning. Participants in IG1.3 will also receive the same content, but all rehearsal elements such as quizzes, tests or the summary of key points at the end of each chapter will be removed from the e-learning environment. Participants in IG2 will receive the same content as participants in the IG1.1, but not within the interactive e-learning environment, but in a non-interactive PDF format. The PDF format will also not include the audio instructions. Participants in the control group will not receive any intervention and will only be referred to the CIS brochure “Your journey through cancer” (84). The brochure, while emphasizing informed decision-making and offering practical advice on medical, psychological, and social aspects of cancer care, does not include guidance on how to search for cancer-related information online.

#### 2.5.2 Development and pre-testing of the intervention

The development of the intervention is based on the innovation and design framework of the Center for eHealth Research and Disease Management's comprehensive roadmap to guide the design and development of an evidence-based eHealth intervention (85).

The pre-testing is divided into three phases. In the first phase, two focus groups of 5–8 cancer patients each will be conducted, and then evaluated. The aim of the focus groups is to answer the following questions: (1) what topics would cancer patients like to be included in the intervention to improve their digital health literacy? (2) What design elements can help patients to improve their digital health literacy? (3) What suggestions do patients have for improving the prototype (86, 87)? During the focus groups, participants will be given a 10-minute PowerPoint presentation of the current content of the intervention to comment on. They will also be presented with different intervention design models to choose from or comment on. The focus groups will be audio recorded and transcribed verbatim. To identify topics that should be included in the intervention, or to identify design elements or suggestions for improvement, the first and second authors will follow an inductive coding process (88). Statements will first be coded and then grouped into categories and subcategories, which will then be named. The thematic analysis will be performed using MAXQDA 2022 (VERBI GmbH). Possible adjustments to the e-learning environment will be discussed by the group of authors.

The second phase is the expert-based cognitive walkthrough (89). Aim of this evaluation is to identify usability issues, including navigation challenges and interface inconsistencies. In addition, the clarity, relevance, and effectiveness of the educational content will be assessed while identifying opportunities for improvement. Five selected internet editors from the CIS, who are very experienced users and have extensive experience in the field of usability testing, will evaluate the e-learning environment (90). The expert-based cognitive walkthrough will be audio recorded to document the experts’ feedback, insights, and suggestions for improvement for further analysis.

In the third phase, a sample of eight cancer patients will participate in a patient-based cognitive walkthrough of the e-learning environment (90, 91). The goal is to gather feedback and understand the user experience by capturing their interaction with the environment, immediate reactions, difficulties, and decision-making processes (90–93). The participants will first complete a short questionnaire that includes HLS-GER 2 screening items (37), age, gender, and primary cancer diagnosis. Participants are instructed to complete the e-learning environment on their own and to think aloud. Thinking aloud will be practiced with participants prior to the start of the study. Patients’ screen activity will be videotaped, their thinking aloud will be audio recorded, and patients will then be interviewed. The audio and video data will be supplemented by handwritten observations by the research assistant. The video and audio recordings will be analyzed independently by two of the authors and coded into themes representing usability issues, content issues, and suggestions for improvement (90). These themes will be discussed by the group of authors and adjustments will be made if necessary.

### 2.6 Outcomes of the main study (RCT)

#### 2.6.1 Primary outcome

The primary outcome is the patient-reported digital health literacy based on the German version of the Digital health literacy Instrument (DHLI) (Supplementary Data Sheet 1) (94, 95), resulting from the mean of the 21 items included. All 21 items are answered on a four-point scale (1 = “very difficult” to 4 = “very easy” and from 1 = “never” to 4 = “often”). A higher mean score indicates higher digital health literacy. The internal consistency of the instrument (Cronbach alpha = .87) was acceptable (35). The questionnaire will be administered at T0, T1 and T2.

#### 2.6.2 Secondary outcomes

In addition to the 21 items of the DHLI, the digital health literacy of the participants will also be measured with six performance-based items of the DHLI (35) - one item each for the sub-skills: operational skills, navigation skills, evaluating reliability, information searching, determining relevance and protecting privacy (Supplementary Data Sheet 2). In the original Dutch version, the performance-based items proved difficult to validate as they did not form a single construct (Cronbach's alpha = .47) (35). No item was created for the subskill of “adding self-generated content” for two reasons. (1) In the original Dutch version, this skill was operationalized as a task in which participants were asked to write a short message to their doctor asking about vaccinations for a trip to Morocco. However, there were no clear standards to evaluate whether the written message was correct or incorrect, leading to potential subjectivity and inconsistency in assessment. (2) The content of our intervention does not directly focus on improving this specific subskill, and therefore, we do not expect meaningful changes in it from baseline to follow-up. Participants are asked to apply the six skills mentioned above (navigation skills, etc.) in a fictitious situation. For example, the performance-based item for measuring navigation skills shows a picture of a screen background (=print screen) with an open Internet browser. Participants are offered five options (multiple choice) to open a new tab in the internet browser. The answers are coded as correct (score = 1) or incorrect (score = 0) and then the scores of all six items are added together to form a total score. The items will differ from the original questionnaire, as these items in the original questionnaire are formulated in Dutch and the questions were designed for patients with rheumatoid arthritis. In addition, there will be different items for navigation and operating skills, depending on which digital device (PC/laptop vs. tablet/smartphone) and operating system (Android vs. iOS and windows vs. macOS) the participants prefer for Internet access. For the piloting of the performance-based items, we created a total of eight items. We created two items each for operational skills and information skills. After piloting, the number of items will be reduced to six. The questionnaire will be administered at T0, T1 and T2.

The German translation of the Reduced Instructional Materials Motivation (RIMMS) will be used to measure participants’ motivation to use the e-learning environment. The RIMMS is a 4-dimensional (attention, relevance, confidence, satisfaction), 12-item short version of the “Instructional Materials Motivation Survey”. The items are rated on a 5-point scale from 1 = “strongly disagree” to 5 = “strongly agree” (96). We will use the resulting mean value across all 12 items for the overall assessment of motivation. The questionnaire was translated from English into German according to a standardized procedure described by the European Organization for Research and Treatment of Cancer (EORTC) Quality of Life Group (97). The RIMMS will be piloted with the performance-based items of the DHLI and will be administered at T1.

E-learning usage data, such as time spent in the e-learning environment and which learning blocks were clicked on, is collected anonymously to measure learner engagement with the e-learning environment in cross-section.

There questionnaires will be used to measure acceptance [Acceptability of Intervention Measure (AIM) questionnaire], appropriateness [Intervention Appropriateness Measure (IAM) questionnaire], and feasibility [Feasibility of Intervention Measure (FIM) questionnaire (98)] of the e-learning environment from the participants’ perspective. The AIM, IAM and FIM questionnaires were translated from English into German according to the standardized procedures of the EORTC Quality of Life Group (97). All three questionnaires contain 4 items that are rated on a 5-point scale from 1 = “disagree” to 5 = “agree” (98). Acceptability, appropriateness and feasibility are presented as the mean value of the four items of each questionnaire, with a higher score indicating greater acceptance, appropriateness and feasibility. The three questionnaires AIM, FIM and IAM will be administered at T1.

Other outcomes used to determine the acceptability of the e-learning environment are the number of discontinuations of the e-learning environment or deviations from the treatment protocol. Participants in the intervention groups will be asked at T1 (1) if they completed all chapters of the e-learning, (2) which chapters they completed, if they did not complete all chapters, and (3) the reasons why they stopped the e-learning environment early (open-ended question).

#### 2.6.3 Participants’ sociodemographic, recruitment and medical characteristics and participants’ internet use

To describe the sample, the following sociodemographic and medical data will be collected at T0: age, sex, marital status, cancer type, self-reported health status, time since cancer diagnosis, educational level, and recruitment route (CIS recruitment route vs. non-CIS recruitment route) into the study.

Participants’ Internet use will be measured at T0 using items on the following topics. These are the frequency of Internet use, preferred digital device used to access the Internet, the preferred mobile phone (IOS, Android or other operating system) or computer or laptop operating system (Windows, macOS or other operating system), and the use of various information services.

#### 2.6.4 Safety endpoints

To rule out protocol deviations, participants in the intervention groups will be asked at T1 whether the content of the intervention was completed by themselves or by someone close to them (e.g., spouse or child). Participants in the control group will be asked at T2 whether they had access to the content of the intervention groups (e.g., through friends/acquaintances in a patient support group).

#### 2.6.5 Piloting the questionnaires

The DHLI, the 7 performance-based items of the DHLI, and the RIMMS will be piloted with 8 patients before the start of the study by means of a three-step test interview (TSTI) (99, 100). It combines observation and questioning techniques to determine how items are interpreted and whether problems occur when completing the questionnaire. In the first phase of the TSTI, the participant will complete the questionnaire while thinking aloud while the interviewer will focus and on observing the participant and taking notes (99). In the second phase, respondents will be interviewed regarding their response behavior, augmenting the data gathered in the first step. In the third phase, patients can share their considerations and opinions about the questionnaire (99, 100). The test is audio-recorded and then transcribed to facilitate evaluation. Analysis will be performed by hand, on item level, except for comments made in step 3 which concern the questionnaire as a whole. Comments and problems will be labelled and subsequently grouped into categories. Coding will be performed independent by two of the authors. For the sub-skills of the performance-based DHLI for which more than one item was created, only one of the two items will be selected for the main study questionnaire. It will be evaluated which item is easier for participants to understand and which item seems to discriminate better between people with low and high digital health literacy.

### 2.7 Sample size calculation

**Figure 2:**
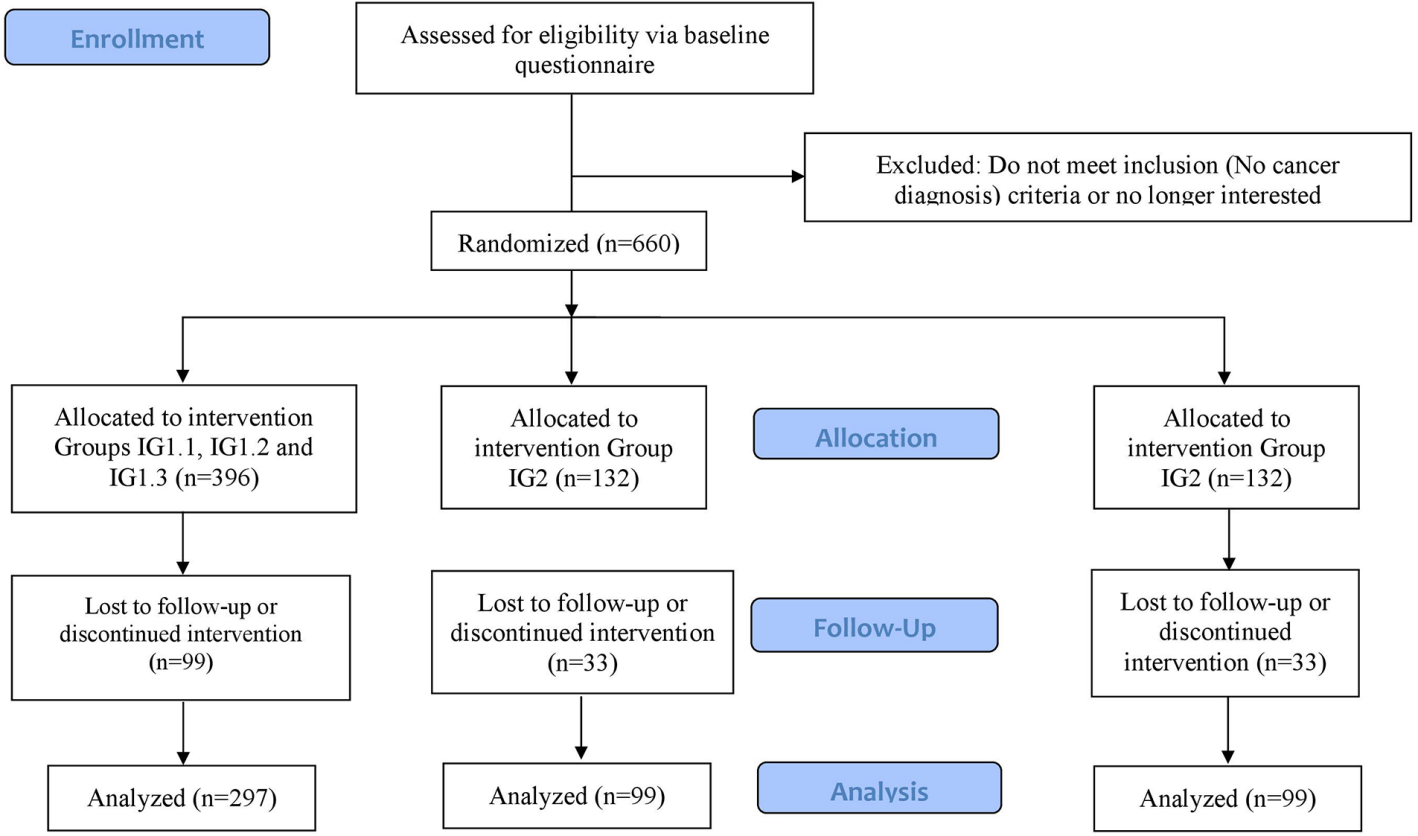
Expected flow chart for participation in the study.

The sample size calculation for the primary hypothesis was calculated using PASS 2008 for a one-way ANOVA. We set the two-sided significance level at 5% and the power at 80%. For the sample size calculation, we expected that at T2 there will be a greater improvement in digital health literacy in the e-learning groups (IG1.1–IG1.3) and IG2 (non-interactive PDF group) compared to the control group. The study by Mitsuhashi (32) found an effect size of Cohen's *d* = 0.25 at 2 weeks. We assume that we will achieve a slightly higher effect size: First, because we involve patients in the design process of the digital learning offerings, and second, because the content of our e-learning environment is presented in a more interactive, persuasive and engaging manner. As mentioned in the introduction, research suggests that online courses with high levels of interactivity lead to higher levels of students’ motivation, improved learning outcomes, and satisfaction than less interactive learning environments (48–51). Furthermore, a systematic review identified a positive correlation between the number of persuasive elements and the effectiveness of web-based interventions (61). In the case number design, we assume an effect size between the e-learning environment (IG1.1, IG1.2 and IG1.3) and the control group and between IG2 and the control group of Cohen's *d* = 0.35. These assumptions correspond to an effect size of *f* = 0.165. The sample size calculation results in 297 participants for the three e-learning environment groups (IG1.1, IG1.2, and IG1.3) and 99 each for IG2 and the control group, for a total of 495 participants (Figure 2).

In the above-mentioned study by Mitsuhashi (47), 6% of participants dropped out in the 2 weeks between baseline and follow-up. In other RCTs involving the promotion of digital health literacy in older adults, dropout rates ranged from 13% to 26% in the period between 2 weeks and 9 months (101–104). With a period of 8 weeks between T0 and T2, we assume a dropout rate of 25%. To compensate for this dropout rate, a total of 660 subjects must be enrolled. 396 people will be equally assigned to the groups IG1.1, IG1.2 and IG1.3 and 132 each for IG2 and the control group.

### 2.8 Allocation and blinding

After completing the online baseline questionnaire, the participants are assigned to one of the following groups according to the randomization sequence stored in REDCap. Participants in each treatment groups will receive an email containing a link tailored to their assigned group: a link to the appropriate e-learning environment (IG1.1–IG1.3), a PDF file (IG2), or a link to the CIS brochure “Your Journey through Cancer” (control group). The randomization sequence was created using the R package “blockrand” (version 1.5) (105) and follows a 1:1:1:1:1 ratio across five groups: IG1.1 (e-learning environment), IG1.2 (e-learning environment without “tunneling” elements), IG1.3 (e-learning environment without “rehearsal” elements), IG2 (non-interactive PDF file), and the control group. Allocation will be conducted using a stratified randomization with variable block lengths. The strata consider both the recruitment route (CIS vs. non-CIS) and the cancer type (breast cancer vs. other cancers). Given that a disproportionate number of inquiries to the CIS come from women (64%) and from patients with breast cancer (39%) (106), the stratification ensures a proportionally representative allocation. To achieve this, randomization is conducted separately for each stratum, ensuring that the distribution of cancer types within the groups reflects the cancer incidence in 2020 (107) in the German population. In 2020, 14.5% of all new cancer diagnoses were breast cancer diagnoses. Two thirds of the planned 660 cancer patients will be recruited through recruitment routes associated with the CIS and one third through recruitment routes such as the outpatient clinics of the UCCH and affiliated patient support groups. The randomization sequence is securely stored and managed within REDCap's automated allocation module, ensuring that neither participants nor recruiting researchers have access to it, and assignments are made only after enrollment to maintain allocation concealment. Blinding will be maintained throughout the duration of the study to increase the objectivity of the results and minimize the influence of participants’ expectations on the study results. Participants in the control group will not receive any intervention and will be referred to the CIS brochure “Your journey through cancer”, which will not include any information on how to search for cancer-related information online. In the data set and in REDCap, the 5 randomization groups are referred to as groups A, B, C, D and E, so that it is not clear to the research staff evaluating the data which intervention the patients received (blinding).

### 2.9 Data collection and management

All data will be collected using the electronic data collection software REDCap (68). The research staff who will create the questionnaires in REDCap and who will export the participants’ data to the statistics software R (version 4.2.1) have been trained in the use of REDCap and will be supported by the REDCap support team of the UKE in case of problems. Data entry in REDCap will be simulated for 10 subjects prior to the start of the study to identify possible problems. All REDCap servers are located in Germany. The data in REDCap is pseudonymized. The list linking the pseudonym to the real person is stored separately on UKE servers and is subject to technical and organizational measures to ensure that the personal data cannot be assigned to an identified or identifiable natural person. The REDCap data are downloaded every 2 weeks to create backup data sets and are also stored on the servers of the UKE. The data will be available only to authors affiliated with the UKE. Due to the characteristics of the study, no data monitoring committee will be included.

Participants will receive an invitation to the 2 and 8 week follow-up via email from the project email address. If they do not complete the questionnaire after the first invitation, they will receive a reminder after 48 h and a second reminder 48 h after the first reminder.

### 2.10 Data analysis

We will use the statistics software R for the statistical analyses. To describe participants’ sociodemographic and medical characteristics, participants’ Internet use, and primary and secondary outcomes at T0, T1, and T2, descriptive statistics such as mean, standard deviation, median, and interquartile range will be calculated for continuous variables. Absolute and relative frequencies will be reported for categorical variables. Descriptive statistics will be reported for the total sample as well as separated by randomization groups.

The evaluation of the primary hypothesis is based on the closed testing principle (108): In the first step, a global test is carried out to determine whether the change in the digital health literacy value from T0 to T2 differs significantly between at least two of the three randomization groups (digital e-learning (IG1.1–IG1.3), non-interactive PDF group, control group). The two-sided significance level is set to 5%. A linear mixed model is calculated to analyze this primary hypothesis. The R package “nlme” will be used for this purpose (109). The dependent variable is the difference between the DHLI value after intervention at T2 and T1, respectively, and the baseline measurement at T0. The randomization group, the follow-up time points after intervention at T1 and T2, the recruitment route (CIS vs. Non-CIS), the cancer type (breast cancer vs. other cancer types) and the interaction between the follow-up time points and the randomization group will be included as fixed factors. To control for differences in baseline DHLI scores, regression to the mean, and to reduce the error variance in the dependent variable (110, 111), and the baseline DHLI value will be included as a covariate. In addition, a random intercept will be estimated for the patients. The global hypothesis shows a significant result if the *p*-value of the global comparison between the three randomization groups at time T2 is smaller than the two-sided significance level.

Only if the result is significant, the pairwise comparisons between the three randomization groups at time T2 will be calculated in the second step using *a priori* contrasts (112) using the R package “emmeans” (113). A pairwise comparison is significant, if the associated *p*-value is smaller than the two-sided significance level. In this way, we keep the overall two-sided type I error at a maximum of 5%. The primary null hypothesis is rejected if either the difference between IG1.1–IG1.3 and the control group and/or between IG2 and the control group is significant. To determine which of the persuasive elements (tunneling, rehearsal) of the e-learning environment influence effectiveness (hypothesis 2), we conduct further pairwise comparisons, again using the R package “emmeans”.

The evaluation of the primary and secondary hypotheses is based on the full analysis set. It is as complete as possible and as similar as possible to the intention-to-treat population. The intention-to-treat population includes all randomized patients belonging to the group to which they were originally randomized, regardless of whether protocol violations are known. As sensitivity analyses, the primary evaluation is repeated in the per-protocol (PP) population. The PP population includes patients without serious protocol violations. We define a serious protocol violation as: (1) Less than 50% of the content of the digital learning offers is processed by participants (is checked with the item on the number of chapters processed at T1); (2) The content is not processed by the participants of IG1.1, IG1.2, IG1.3 and IG2 themselves, but by a close person (e.g., spouse or child) (checked by an item at T1); (3) Participants in the control group are given access to the learning opportunities of the intervention groups, e.g., via patient friends in self-help groups (checked by item at T2); (4) Participants who report that they do not have cancer. In addition, protocol deviations reported by the participants in the REDCap data set are listed and forwarded to the UKE research assistant, who blinds them (he knows neither the group affiliation nor the outcome values of the participants) and classifies them into severe and less severe protocol deviations.

In the full analysis set, we will not replace missing data. However, as a further sensitivity analysis, the primary analysis is performed with a multiply imported data set. For the imputation, we will use the “Amelia II” package (version 1.8.1 in R (114)) which allows us to generate 100 imputations of missing values using a bootstrapping-based algorithm. We will use the Amelia package to impute data for missing values of the DHLI at T2; there should be no missing values at T0 in a FAS population. The DHLI values at T2 will be defined as continuous variables and we will define logical boundaries (min = 1, max = 4). We will pool the results of the linear mixed model for the 100 imputed datasets based on Rubin's rules (115).

The third research question is evaluated analogously to the primary hypothesis using linear mixed models. The dependent variable is changed from the DHLI value to the total score of the performance based items of the DHLI.

A mediation model is used to analyze whether motivation mediates the association between randomization groups and the change in digital health literacy from T0 to T2 (research question 5). The mediation model is calculated with patient motivation (mean RIMMS score) as the mediator; the intervention group as the independent variable. The dependent variable is the difference in digital health literacy from T0 to T2. The mediation analysis is performed based on the model of Hayes (116).

The results of the RIMMS, AIM, IAM and FIM will be presented and interpreted descriptively to investigate user-friendliness, appropriateness and feasibility from the patient perspective (research question 6). To evaluate whether the e-learning environment is perceived as more user-friendly, appropriate and feasible that the non-interactive PDF file (research question 7), we will use Analysis of Variance (ANOVA) tests.

A statistical analysis plan for all intended analyses will be finalized before unblinding.

## 3 Discussion

The primary objective of this study is to investigate whether cancer patients can improve their digital health literacy over an 8-week period using either an interactive e-learning environment or a non-interactive PDF file compared to a control group. This study is motivated by the recognition of the increasing importance of digital health literacy in empowering patients to navigate complex healthcare systems and make informed decisions about their health. In particular, cancer patients with less common cancers, such as small bowel or anal cancers, can benefit significantly from improved digital health literacy due to the lack of online resources tailored to their specific conditions. Digital health literacy is part of health literacy, which has been found to be insufficient in the populations of many countries (37, 117–119). Poor digital health literacy can negatively influence health by limiting individuals’ ability to access, understand, and use online health information effectively. This can lead to misinformed health decisions, non-adherence to medical instructions, and delayed or inappropriate treatment-seeking behaviors, ultimately resulting in poorer health outcomes (120–122). Promotion of health literacy has become an integral part of European health strategies, of global activities of the United Nations, as well of political strategies or national plans of many countries (123). In contrast to the long-term goals of these national plans, this intervention study aims to improve the digital health literacy of cancer patients, who have a high unmet need for information (1), in a timely manner.

If the study results show that the e-learning environment has a positive impact on cancer patients’ digital health literacy compared to a control group, it would be made freely available to all cancer patients through the CIS website. Caregivers, who play a crucial role in supporting cancer patients, would also have access to the e-learning environment, allowing them to enhance their own understanding and better assist the patients in their care. The wide reach of the CIS website, with approximately 7.7 m visitors in 2023, means that many cancer patients can be reached. The e-learning environment could empower patients to be more involved in their medical decision-making (10, 11), be better informed about their disease (5), and may even positively influence their health-related QoL (124). If adapted accordingly, patients with other chronic diseases or healthy people could also benefit from the e-learning environment. Since cancer patients, patients with other chronic diseases such as rheumatoid arthritis, and healthy people seem to have the same problems when searching for health information on the Internet (34, 36, 125), the latter two groups could also potentially improve their digital health literacy by using the e-learning environment.

There are two main concerns regarding the generalizability of the study results. Two-thirds of the participants are recruited through CIS-related recruitment routes, and a disproportionate number of inquiries to the CIS come from women (64%), and from patients with breast cancer (39%) (106). Although there is no evidence that cancer type has an impact on the primary outcome of digital health literacy (30), we addressed this potential bias by implementing a proportionally stratified randomization strategy in relation to the cancer incidence in 2020 (107) in the German population. Another concern relates to the level of digital health literacy of the participants in the study, especially those who will be recruited through the CIS recruitment route. Given that patients using the CIS platform have a higher level of education compared to the average German cancer patient population, there may be a risk of sampling bias towards individuals with higher digital health literacy. To minimize this bias, a complementary recruitment strategy will be employed in which one-third of participants will be recruited offline from outpatient clinics. By diversifying recruitment routes, we aim to obtain a more representative sample of cancer patients with varying levels of digital health literacy.

The DHLI - the primary outcome - has been validated in different languages and populations (126–132) within the last 5 years and has been translated and used in German-speaking populations (71, 94, 95), but it has not been validated in German, which is a limitation of our study. Nevertheless, we prefer the DHLI to the more commonly used eHealth literacy scale (eHEALS) (33), because we assume that searching for cancer-related information requires different skills that are better measured by the multidimensional DHLI than by the one-dimensional eHEALS. In addition, study results also show that the DHLI has stronger positive correlations with certain factors, such as Internet use for health information and higher educational attainment, than the eHEALS (133). Furthermore, we will pilot test the DHLI with the TSTI using 8 cancer patients to determine how the items are interpreted and if there are any problems in completing the questionnaire. In addition, a validation of the questionnaire using the data from the RCT is planned.

The second limitation of this study is the exclusive use of the German language. This limitation is particularly significant in the context of the diverse population in Germany, where approximately 15% of households primarily speak a language other than German, with Turkish being the most commonly spoken language among this group (134). This language barrier may limit the accessibility and applicability of the study to non-German speaking patients, potentially excluding a portion of the population that could benefit from the intervention. Nevertheless, we should not overlook the fact that the use of multiple languages in the initial study phase could introduce confounding factors related to translation differences, which may complicate the interpretation of results. By focusing on a single language, we aim to maintain the internal validity of the study, which is critical for drawing accurate and reliable conclusions.

The third limitation is the absence of an explicit behavioral theory as a foundation for the intervention. Although the intervention incorporates persuasive design element of the PSD model, it does not explicitly incorporate additional behavioral theories during its development. Behavioral theories could have provided a stronger theoretical foundation to guide the intervention's design and implementation. This represents a limitation in the conceptual framework of the study. Related studies should consider explicitly integrating behavioral theories to enhance the rationale and effectiveness of the approach.

## Data Availability

The original contributions presented in the study are included in the article/Supplementary Material. Further inquiries can be directed to the corresponding author. The study data will be made available to other scientists via the online open access repository Figshare.
