# Supplementary material for: Development and effectiveness evaluation of an interactive e-learning environment to enhance digital health literacy in cancer patients: study protocol for a randomized controlled trial
Source: Front Digit Health. 2025 Jan 24;7:1455143. doi: 10.3389/fdgth.2025.1455143 (PMC11802532; doi:10.3389/fdgth.2025.1455143)
Supplement: Supplementary file 1 [file Datasheet1.pdf]

Supplementary Material 1.

Digital Health Literacy Instrument

Im folgenden Abschnitt geht es um Ihre Einschätzung der eigenen Kompetenz das Internet zu nutzen.

**1** Wenn Sie im Internet nach Informationen zu Gesundheitsthemen suchen wie einfach oder schwierig ist es für Sie, ...	sehr einfach	einfach	schwierig	sehr schwierig
... die Tastatur eines Computers zu benutzen?	☐1	☐2	☐3	☐4
... die Computer-Maus zu benutzen?	☐1	☐2	☐3	☐4
… die Schaltflächen oder Links und Hyperlinks auf Websites zu benutzen?	☐1	☐2	☐3	☐4

**2** Wenn Sie im Internet nach Informationen zu Gesundheitsthemen suchen, wie einfach oder schwierig ist es für Sie …	sehr einfach	einfach	schwierig	sehr schwierig
... eine Auswahl aus allen Informationen zu treffen, die Sie finden?	☐1	☐2	☐3	☐4
... die richtigen Begriffe oder Suchanfragen zu verwenden, um die Informationen zu finden, die Sie suchen?	☐1	☐2	☐3	☐4
… genau die Informationen zu finden, die Sie suchen?	☐1	☐2	☐3	☐4

**3** Wenn Sie im Internet nach Informationen zu Gesundheitsthemen suchen wie einfach oder schwierig ist es für Sie, ...	sehr einfach	einfach	schwierig	sehr schwierig
... zu beurteilen, ob die Informationen zuverlässig sind oder nicht?	☐1	☐2	☐3	☐4
... zu beurteilen, ob Informationen mit kommerziellem Interesse geschrieben worden sind (etwa von Personen oder Unternehmen, die ein Produkt verkaufen)?	☐1	☐2	☐3	☐4
… verschiedene Websites daraufhin zu überprüfen, ob sie die gleichen Informationen enthalten?	☐1	☐2	☐3	☐4

4 Wenn Sie im Internet nach Informationen zu Gesundheitsthemen suchen wie einfach oder schwierig ist es für Sie, ...	sehr ein-fach	einfach	schwierig	sehr schwierig
... die gefundenen Informationen zu nutzen, um Entscheidungen über Ihre Gesundheit zu treffen (etwa über Ernährung, Medikamente oder um zu entscheiden, ob Sie die Meinung eines Arztes einholen wollen)?	☐1	☐2	☐3	☐4
... die gefundenen Informationen in Ihrem Alltag anzuwenden?	☐1	☐2	☐3	☐4
… zu beurteilen, ob die Informationen, die Sie gefunden haben, auf Sie zutreffen?	☐1	☐2	☐3	☐4

5 Wenn Sie im Internet nach Gesundheitsinformationen suchen, wie oft kommt es vor, dass Sie…	nie	selten	manchmal	häufig
... den Überblick verlieren, wo Sie sich auf einer Website oder im Internet befinden?	☐1	☐2	☐3	☐4
... nicht wissen, wie Sie zu einer vorherigen Seite zurückkommen können?	☐1	☐2	☐3	☐4
… auf etwas klicken und etwas anderes zu sehen bekommen, als Sie erwartet haben?	☐1	☐2	☐3	☐4

6 Wenn Sie eine gesundheitsbezogene Nachricht schreiben, wie einfach oder schwierig ist es für Sie, ...	sehr ein-fach	einfach	schwierig	sehr schwierig
... Ihre Frage oder Ihr gesundheitliches Anliegen klar zu formulieren?	☐1	☐2	☐3	☐4
... Ihre Meinung, Gedanken oder Gefühle schriftlich auszudrücken?	☐1	☐2	☐3	☐4
... Ihre Nachricht so zu schreiben, dass andere Personen genau verstehen, was Sie meinen?	☐1	☐2	☐3	☐4

7 Wenn Sie eine Nachricht rund um das Thema Gesundheit in einem öffentlichen Forum oder in sozialen Medien veröffentlichen, wie oft ...				
	häufig	manchmal	selten	nie
... finden Sie es schwierig zu beurteilen, wer mitlesen kann?	☐1	☐2	☐3	☐4
... geben Sie (absichtlich oder unabsichtlich) private Informationen über sich weiter (wie Name oder Adresse)?	☐1	☐2	☐3	☐4
… geben Sie (absichtlich oder unabsichtlich) private Informationen einer anderen Person weiter?	☐1	☐2	☐3	☐4
