# Supplementary material for: Development and effectiveness evaluation of an interactive e-learning environment to enhance digital health literacy in cancer patients: study protocol for a randomized controlled trial
Source: Front Digit Health. 2025 Jan 24;7:1455143. doi: 10.3389/fdgth.2025.1455143 (PMC11802532; doi:10.3389/fdgth.2025.1455143)
Supplement: Supplementary file 2 [file Datasheet2.pdf]

Supplementary Material 2

DHLI performance based items

Version for Windows users:

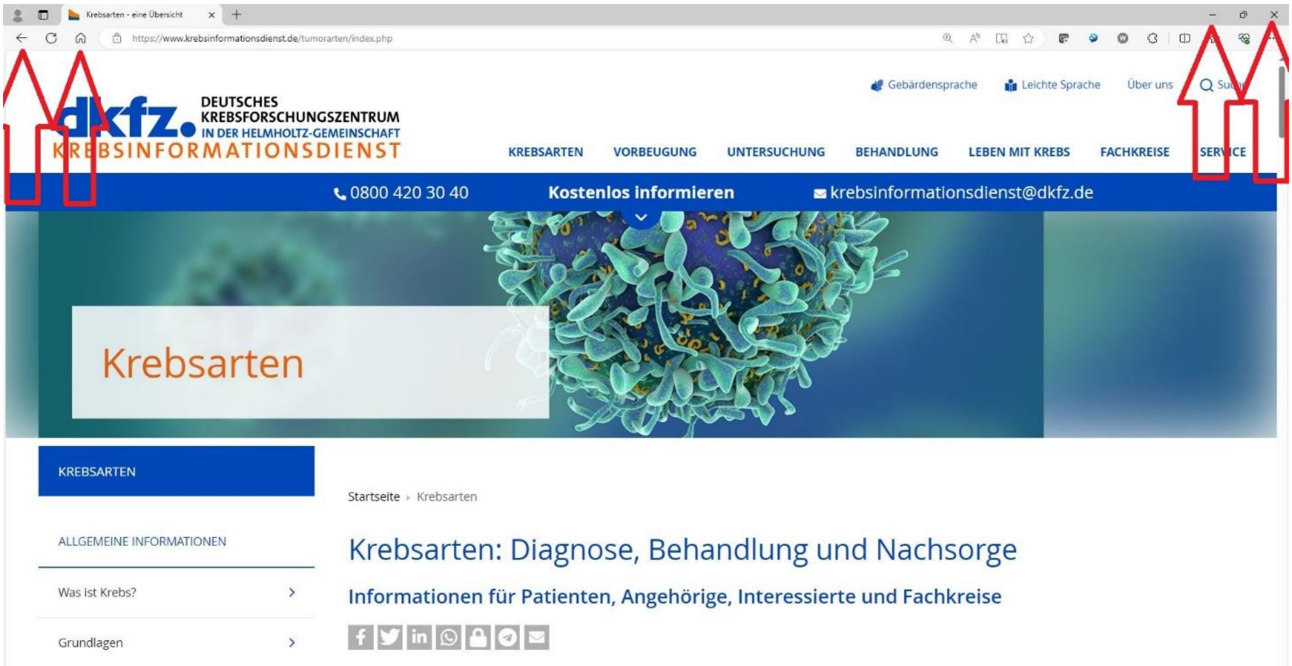

Sie sind über die Startseite des Krebsinformationsdienst auf die folgende Seite gelangt und möchten jetzt wieder zurück auf die Startseite. Wie kommen Sie zurück auf die Startseite?

☐1 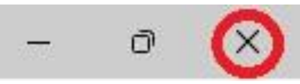

☐2 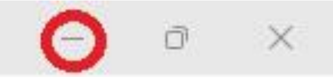

☐3 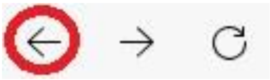

☐4 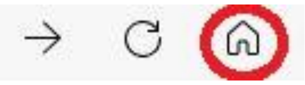

☐5 Ich weiß es nicht

Ihr Computer schreibt nur noch in Großbuchstaben. Was müssen Sie tun, um das wieder rückgängig zu machen?

☐1 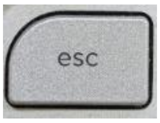

☐2 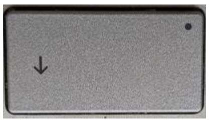

☐3 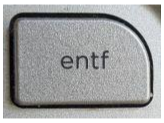

☐4 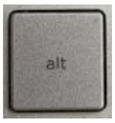

☐5 Ich weiß es nicht

Sie möchten sich von einer Internetseite Informationen kopieren. Wie machen Sie das?

☐1 Die gewünschten Informationen markieren und die Tasten „alt gr“ und „c“

☐2 Die gewünschten Informationen markieren und die Tasten „Strg“ und „c

☐3 Die gewünschten Informationen markieren und die Tasten „Strg“ und „k“

☐4 Die gewünschten Informationen markieren und die Tasten „alt gr“ und „k“

☐5 Ich weiß es nicht

frontiers

Sie recherchieren bei Google Ernährungstipps zu ihrer Krebserkrankung. Bei welchen Seiten ist ein kommerzielles Interesse zu vermuten. Kreuzen sie an. (Es dürfen mehr als eine Antwortmöglichkeit ausgewählt werden).

☐1

Deutsche Krebsgesellschaft
https://www.krebsgesellschaft.de › basis-informationen...

Ernährung und Krebs

Erfahren Sie mehr über gesunde **Ernährung** zur Krebsprävention und welche Schadstoffe im Essen Sie meiden sollten um **Krebs** vorzubeugen.

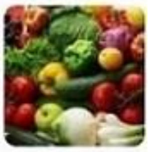

☐2

Gesponsert

Naturheilkompass
https://www.naturheilkompass.de › bestrahlung

Richtige Ernährung bei Krebs - Das können Sie selbst tun

Strahlentherapie natürlich unterstützen. Infos & Empfehlungen für Betroffene & Angehörige. Dr. Hüther informiert Sie über Nebenwirkungen und Therapieunterstützung bei Bestrahlung.

Die richtige Ernährung · Die 4-Schritte-Anleitung · Chemo-Nebenwirkungen

---

☐3

Krebsinformationsdienst
https://www.krebsinformationsdienst.de › iblatt PDF

Informationsblatt: Ernährung bei Krebs

Page 1. **ERNÄHRUNG BEI KREBS**: Was ist wichtig? y Eine individuell angepasste Ernährung kann die Ver- träglichkeit einer Krebsbehandlung und damit die Le-.

☐4

Gesponsert

Stärker gegen Krebs
https://www.staerkergegenkrebs.de

Ernährungsberater finden - in Ihrer Nähe & vergleichen

Erfahren Sie mehr zu dem Einfluss der **Ernährung** auf Ihre **Krebserkrankung**. Finden Sie Ihren Ernährungsberater vor Ort. Inklusive Informationen & Kontaktmöglichkeit. Therapien.

Was zahlen Krankenkassen · Unterstützung bei Krebs · Nebenwirkungen Lindern

---

☐5 Ich weiß es nicht

Sie suchen im Internet nach Informationen zu „Linderungsmöglichkeiten von krebsassoziierter Fatigue. Fatigue ist ein Zustand außerordentlicher Müdigkeit und mangelnder Energiereserven, der selbst nach angemessenen Ruhepausen nicht verschwindet. Welche der Links beinhaltet mit hoher Wahrscheinlichkeit die Informationen, zu dem Thema?

☐1

gesundheitsforschung-bmbf.de
https://www.gesundheitsforschung-bmbf.de › erschopf...

Erschöpfungssyndrom bei Krebskranken

Fatigue heißt diese besondere Form der Erschöpfung bei **Krebs**. Sie belastet viele Patienten auch noch Jahre nach Abschluss einer Tumortherapie. Wissenschaftler ...

☐2

Deutsche Krebsgesellschaft
https://www.krebsgesellschaft.de › fatigue-bei-krebs

Tumor-assoziierte Fatigue

Tritt eine anhaltende Erschöpfung im Zusammenhang mit einer **Krebserkrankung** auf, wird sie als Tumor-assoziierte **Fatigue** bezeichnet. Typisch für **Fatigue** sind ...

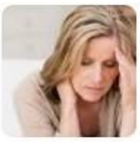

☐3

Deutsche Krebshilfe
https://www.krebshilfe.de › ... › Mit Krebs leben

Fatigue (Chronische Müdigkeit bei Krebs)

Fatigue ist eine langanhaltende, chronische Müdigkeit, die während und zum Teil noch längere Zeit nach der Behandlung einer **Krebserkrankung** besteht und auch ...

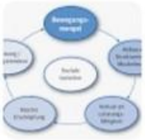

☐4

Krebsinformationsdienst
https://www.krebsinformationsdienst.de · vor 3 Jahren

Fatigue bei Krebs: Was hilft gegen extreme Müdigkeit?

Entspannungsübungen: Yoga, Massagen oder progressive Muskelentspannung können eine **Fatigue lindern**. Betroffene sollten überlegen, von welchem ...

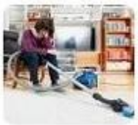

☐5 Ich weiß es nicht

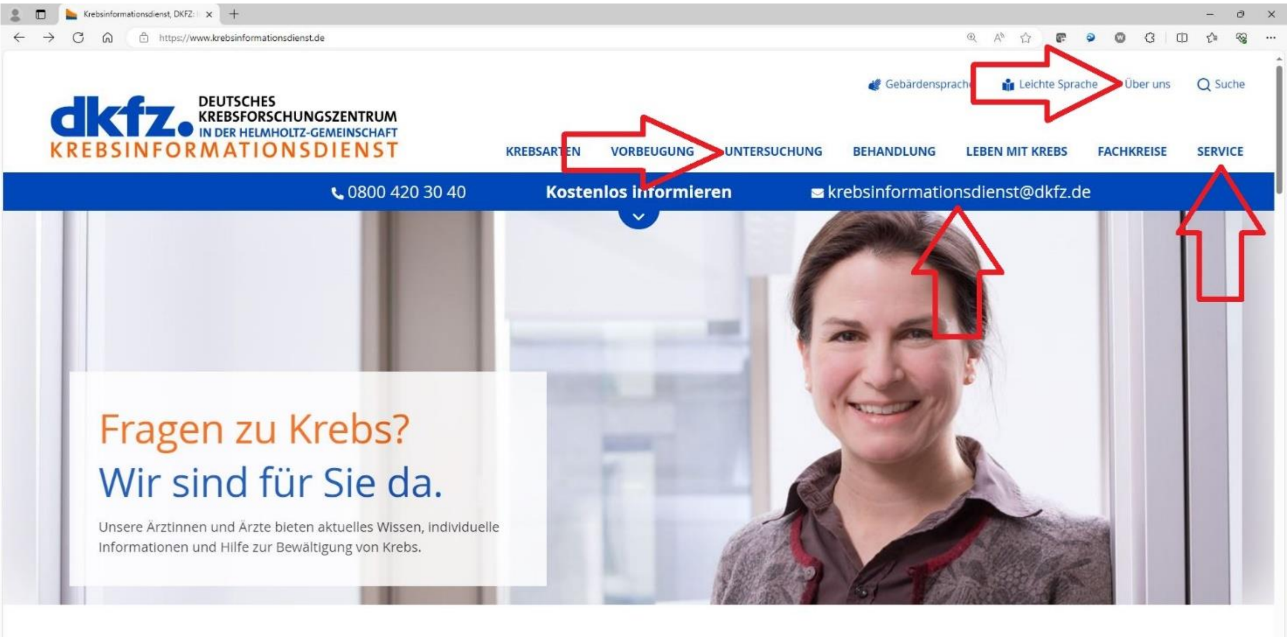

Stellen Sie sich vor Sie sind auf der Website des Krebsinformationsdienst und möchten wissen, was genau der Krebsinformationsdienst macht und wer dahinter steht. Auf welche Schaltfläche klicken Sie?

☐1 Über uns

☐2 UNTERSUCHUNG

☐3 SERVICE

☐4 krebsinformationsdienst@dkfz.de

☐5 Ich weiß es nicht

Stellen Sie sich vor, dass sie als Krebspatient*in an chronischer Müdigkeit leiden. Ihr Partner vermutet, dass Sie an krebsassoziierter Fatigue leiden. Fatigue ist ein Zustand außerordentlicher Müdigkeit und mangelnder Energiereserven, der selbst nach angemessenen Ruhepausen nicht verschwindet. Sie möchten die genauen Symptome von krebsassoziierter Fatigue herausfinden. Eine Google-Suche führt sie zu den unten stehenden Ergebnissen. Welches dieser Ergebnisse würde Ihnen am ehesten eine korrekte und zuverlässige Antwort geben?

☐1
DocCheck Flexikon
https://flexikon.doccheck.com › Fatigue
Fatigue
Fatigue ist ein Syndrom (eine Ansammlung unterschiedlicher Symptome), das als Begleiterscheinung verschiedener chronischer Krankheiten auftritt, ...

☐2
BILD.de
https://www.bild.de › Leben & Wissen › Medizin
Müdigkeit, Gewichtsverlust: Diese Krebs-Symptome ...
12.06.2012 — Sind Sie immer **müde**? Haben Sie abgenommen oder gar unerklärliche Schmerzen? Dann sollten Sie schleunigst zum Arzt gehen. Es könnte **Krebs** ...

☐3
Deutsche Krebsgesellschaft
https://www.krebsgesellschaft.de › onko-internetportal
Fatigue bei Krebs - Überblick
Übermäßige Schonung, so der aktuelle wissenschaftliche Stand, hat keinen positiven Effekt auf die **Fatigue-Symptome**. ... **krebsassoziierte-fatigue**-chronische ...

☐4
Rheuma-Liga
https://www.rheuma-liga.de › rheuma › krankheitsbilder
Fatigue-Syndrom: Kampf gegen Müdigkeit und Erschöpfung
Viele rheumatische Erkrankungen gehen mit **Fatigue** einher. Die Ursachen für die andauernde Erschöpfung sind noch nicht geklärt.

☐5 Ich weiß es nicht

Sie sind in einem Forum für Krebspatienten und lesen folgende 3 Beiträge. Welcher dieser fiktiven Beiträge verstößt gegen die Privatsphäre und verletzt den Datenschutz?

☐ 1 Hallo an Alle,
Meine Mutter heißt Marie Schäfer und ist 56 Jahre alt. Vor einem halben Jahr hat sie die Diagnose Brustkrebs bekommen und wird zurzeit mit Chemo therapiert. Sie ist bei Herrn Dr. Schneider im Klinikum Bremen Mitte in Behandlung. Hat jemand dort schon Erfahrung gesammelt?

☐ 2 Moin moin,
Hat jemand hier Erfahrungen mit alternativen Heilpraktiken in Bezug auf Darmkrebs gemacht?
LG Peter

☐ 3 Liebe Community,
ich hoffe, es geht euch allen so gut wie möglich. Ich wollte mich kurz vorstellen und meinen Weg teilen, um Unterstützung zu finden und vielleicht auch anderen Mut zu machen.
Vor einigen Wochen erhielt ich die Diagnose Krebs. Ich bin entschlossen, diesen Kampf anzunehmen und positiv voranzuschreiten. Dabei würde ich gerne von euren Erfahrungen hören, Tipps zur Bewältigung von Nebenwirkungen erhalten und auch einfach nur ein offenes Ohr finden.
Vielen Dank für eure Zeit und Unterstützung. Gemeinsam sind wir stärker!
Liebe Grüße, Sandra

☐ 4 Ich weiß es nicht

Version for android users:

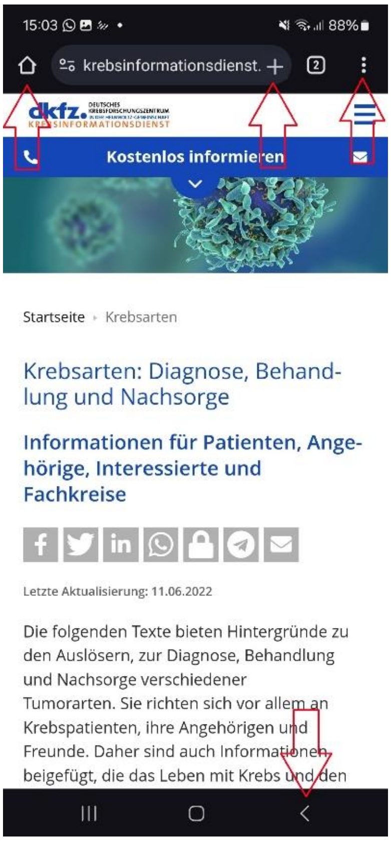

Sie sind über die Startseite des Krebsinformationsdienst auf die folgende Seite gelangt und möchten jetzt wieder zurück auf die Startseite. Wie kommen Sie zurück auf die Startseite?

☐1 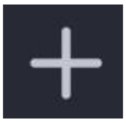

☐2 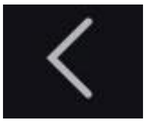

☐3 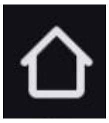

☐4 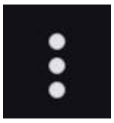

☐5 Ich weiß es nicht

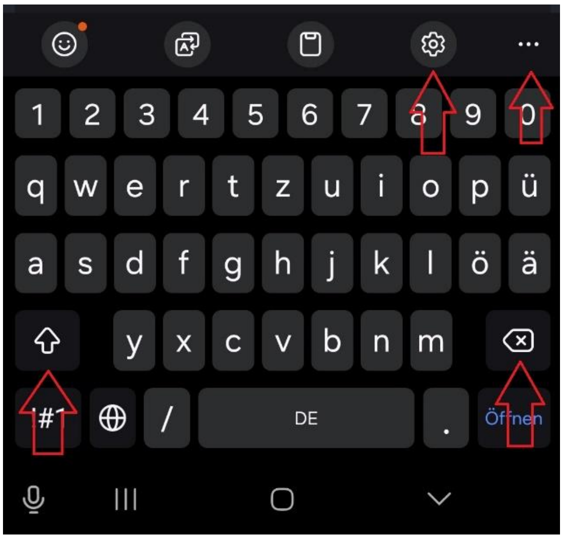

Ihr Handy schreibt nur noch in Großbuchstaben. Was müssen Sie tun, um dies rückgängig zu machen?

☐1 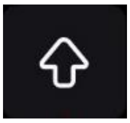

☐2 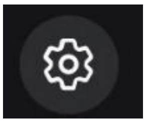

☐3 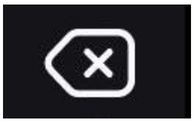

☐4 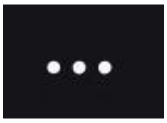

☐5 Ich weiß es nicht

Sie möchten sich von einer Internetseite Informationen kopieren. Wie machen Sie das?

- ☐1 Den Finger lange auf die gewünschten Informationen gedrückt halten und im geöffneten Fenster auf „kopieren“ klicken
- ☐2 Ich mache ein Foto mit einem anderen Handy
- ☐3 Den Finger lange auf die gewünschten Informationen gedrückt halten und im geöffneten Fenster auf „Teilen“ klicken
- ☐4 Ich mache einen Screenshot
- ☐5 Ich weiß es nicht

Sie recherchieren bei Google Ernährungstipps zu ihrer Krebserkrankung. Bei welchen Seiten ist ein kommerzielles Interesse zu vermuten. Kreuzen sie an. (Es dürfen mehr als eine Antwortmöglichkeit ausgewählt werden).

☐1

Deutsche Krebsgesellschaft
https://www.krebsgesellschaft.de › basis-informationen...

Ernährung und Krebs

Erfahren Sie mehr über gesunde **Ernährung** zur Krebsprävention und welche Schadstoffe im Essen Sie meiden sollten um **Krebs** vorzubeugen.

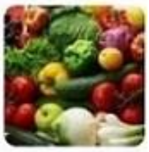

☐2

Gesponsert

Naturheilkompass
https://www.naturheilkompass.de › bestrahlung

Richtige Ernährung bei Krebs - Das können Sie selbst tun

Strahlentherapie natürlich unterstützen. Infos & Empfehlungen für Betroffene & Angehörige. Dr. Hüther informiert Sie über Nebenwirkungen und Therapieunterstützung bei Bestrahlung.

Die richtige Ernährung · Die 4-Schritte-Anleitung · Chemo-Nebenwirkungen

---

☐3

Krebsinformationsdienst
https://www.krebsinformationsdienst.de › iblatt PDF

Informationsblatt: Ernährung bei Krebs

Page 1. **ERNÄHRUNG BEI KREBS**: Was ist wichtig? y Eine individuell angepasste Ernährung kann die Ver- träglichkeit einer Krebsbehandlung und damit die Le-.

☐4

Gesponsert

Stärker gegen Krebs
https://www.staerkergegenkrebs.de

Ernährungsberater finden - in Ihrer Nähe & vergleichen

Erfahren Sie mehr zu dem Einfluss der **Ernährung** auf Ihre **Krebserkrankung**. Finden Sie Ihren Ernährungsberater vor Ort. Inklusive Informationen & Kontaktmöglichkeit. Therapien.

Was zahlen Krankenkassen · Unterstützung bei Krebs · Nebenwirkungen Lindern

---

☐5 Ich weiß es nicht

Sie suchen im Internet nach Informationen zu „Linderungsmöglichkeiten von krebsassoziierter Fatigue. Fatigue ist ein Zustand außerordentlicher Müdigkeit und mangelnder Energiereserven, der selbst nach angemessenen Ruhepausen nicht verschwindet. Welche der Links beinhaltet mit hoher Wahrscheinlichkeit die Informationen, zu dem Thema?

☐ 1

gesundheitsforschung-bmbf.de
https://www.gesundheitsforschung-bmbf.de › erschopf...

Erschöpfungssyndrom bei Krebskranken

Fatigue heißt diese besondere Form der Erschöpfung bei **Krebs**. Sie belastet viele Patienten auch noch Jahre nach Abschluss einer Tumortherapie. Wissenschaftler ...

☐ 2

Deutsche Krebsgesellschaft
https://www.krebsgesellschaft.de › fatigue-bei-krebs

Tumor-assoziierte Fatigue

Tritt eine anhaltende Erschöpfung im Zusammenhang mit einer **Krebserkrankung** auf, wird sie als Tumor-assoziierte **Fatigue** bezeichnet. Typisch für **Fatigue** sind ...

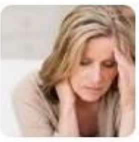

☐ 3

Deutsche Krebshilfe
https://www.krebshilfe.de › ... › Mit Krebs leben

Fatigue (Chronische Müdigkeit bei Krebs)

Fatigue ist eine langanhaltende, chronische Müdigkeit, die während und zum Teil noch längere Zeit nach der Behandlung einer **Krebserkrankung** besteht und auch ...

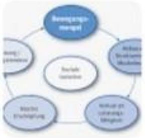

☐ 4

Krebsinformationsdienst
https://www.krebsinformationsdienst.de · vor 3 Jahren

Fatigue bei Krebs: Was hilft gegen extreme Müdigkeit?

Entspannungsübungen: Yoga, Massagen oder progressive Muskelentspannung können eine **Fatigue lindern**. Betroffene sollten überlegen, von welchem ...

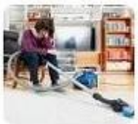

☐ 5 Ich weiß es nicht

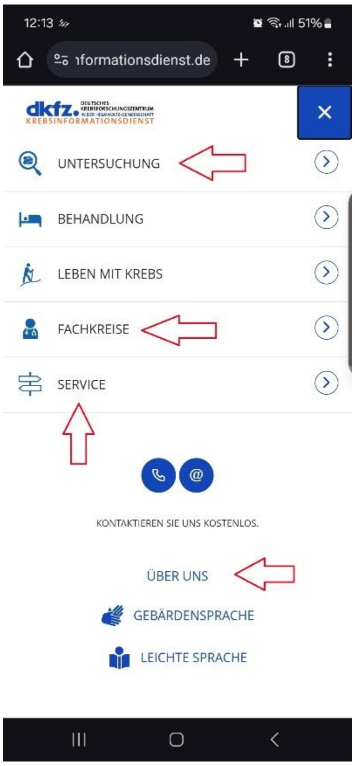

Stellen Sie sich vor Sie sind auf der Website des Krebsinformationsdienst und möchten wissen, was genau der Krebsinformationsdienst macht und wer dahinter steht. Auf welche Schaltfläche klicken Sie?

☐1 UNTERSUCHUNG

☐2 FACHKREISE

☐3 ÜBER UNS

☐4 SERVICE

☐5 Ich weiß es nicht

Stellen Sie sich vor, dass sie als Krebspatient*in an chronischer Müdigkeit leiden. Ihr Partner vermutet, dass Sie an krebsassoziierter Fatigue leiden. Fatigue ist ein Zustand außerordentlicher Müdigkeit und mangelnder Energiereserven, der selbst nach angemessenen Ruhepausen nicht verschwindet. Sie möchten die genauen Symptome von krebsassoziierter Fatigue herausfinden. Eine Google-Suche führt sie zu den unten stehenden Ergebnissen. Welches dieser Ergebnisse würde Ihnen am ehesten eine korrekte und zuverlässige Antwort geben?

☐1

DocCheck Flexikon
https://flexikon.doccheck.com › Fatigue

Fatigue

Fatigue ist ein Syndrom (eine Ansammlung unterschiedlicher Symptome), das als Begleiterscheinung verschiedener chronischer Krankheiten auftritt, ...

☐2

BILD.de
https://www.bild.de › Leben & Wissen › Medizin

Müdigkeit, Gewichtsverlust: Diese Krebs-Symptome ...

12.06.2012 — Sind Sie immer **müde**? Haben Sie abgenommen oder gar unerklärliche Schmerzen? Dann sollten Sie schleunigst zum Arzt gehen. Es könnte **Krebs** ...

☐3

Deutsche Krebsgesellschaft
https://www.krebsgesellschaft.de › onko-internetportal

Fatigue bei Krebs - Überblick

Übermäßige Schonung, so der aktuelle wissenschaftliche Stand, hat keinen positiven Effekt auf die **Fatigue-Symptome**. ... **krebsassoziierte-fatigue**-chronische ...

☐4

Rheuma-Liga
https://www.rheuma-liga.de › rheuma › krankheitsbilder

Fatigue-Syndrom: Kampf gegen Müdigkeit und Erschöpfung

Viele rheumatische Erkrankungen gehen mit **Fatigue** einher. Die Ursachen für die andauernde Erschöpfung sind noch nicht geklärt.

☐5 Ich weiß es nicht

Sie sind in einem Forum für Krebspatienten und lesen folgende 3 Beiträge. Welcher dieser fiktiven Beiträge verstößt gegen die Privatsphäre und verletzt den Datenschutz?

☐1 Hallo an Alle,
Meine Mutter heißt Marie Schäfer und ist 56 Jahre alt. Vor einem halben Jahr hat sie die Diagnose Brustkrebs bekommen und wird zurzeit mit Chemo therapiert. Sie ist bei Herrn Dr. Schneider im Klinikum Bremen Mitte in Behandlung. Hat jemand dort schon Erfahrung gesammelt?

☐2 Moin moin,
Hat jemand hier Erfahrungen mit alternativen Heilpraktiken in Bezug auf Darmkrebs gemacht?
LG Peter

☐3 Liebe Community,
ich hoffe, es geht euch allen so gut wie möglich. Ich wollte mich kurz vorstellen und meinen Weg teilen, um Unterstützung zu finden und vielleicht auch anderen Mut zu machen.
Vor einigen Wochen erhielt ich die Diagnose Krebs. Ich bin entschlossen, diesen Kampf anzunehmen und positiv voranzuschreiten. Dabei würde ich gerne von euren Erfahrungen hören, Tipps zur Bewältigung von Nebenwirkungen erhalten und auch einfach nur ein offenes Ohr finden.
Vielen Dank für eure Zeit und Unterstützung. Gemeinsam sind wir stärker!
Liebe Grüße, Sandra

☐4 Ich weiß es nicht

Version for IOS users:

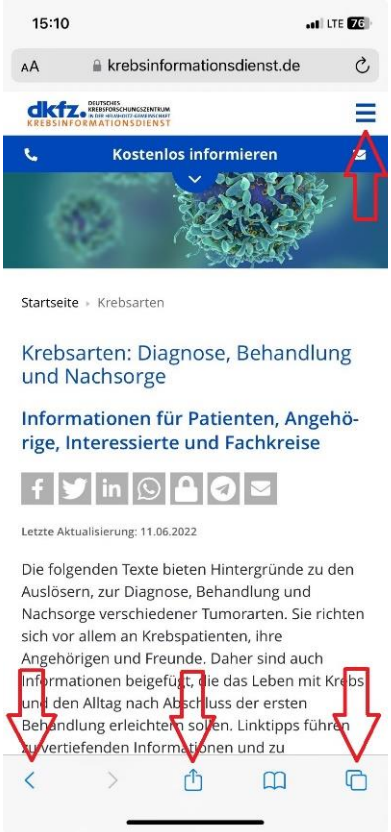

Sie sind über die Startseite des Krebsinformationsdienst auf die folgende Seite gelangt und möchten jetzt wieder zurück auf die Startseite. Wie kommen Sie zurück auf die Startseite?

☐1 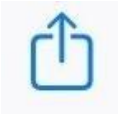

☐2 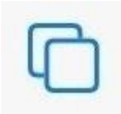

☐3 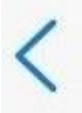

☐4 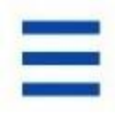

☐5 Ich weiß es nicht

Sie möchten sich von einer Internetseite Informationen kopieren. Wie machen Sie das?

☐1	Den Finger lange auf die gewünschten Informationen gedrückt halten und im geöffneten Fenster auf „kopieren“ klicken
☐2	Ich mache ein Foto mit einem anderen Handy
☐3	Den Finger lange auf die gewünschten Informationen gedrückt halten und im geöffneten Fenster auf „Teilen“ klicken
☐4	Ich mache einen Screenshot
☐5	Ich weiß es nicht

Sie recherchieren bei Google Ernährungstipps zu ihrer Krebserkrankung. Bei welchen Seiten ist ein kommerzielles Interesse zu vermuten. Kreuzen sie an. (Es dürfen mehr als eine Antwortmöglichkeit ausgewählt werden).

☐1

Deutsche Krebsgesellschaft
https://www.krebsgesellschaft.de › basis-informationen...

Ernährung und Krebs

Erfahren Sie mehr über gesunde **Ernährung** zur Krebsprävention und welche Schadstoffe im Essen Sie meiden sollten um **Krebs** vorzubeugen.

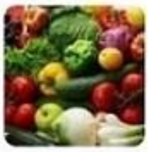

☐2

Gesponsert

Naturheilkompass
https://www.naturheilkompass.de › bestrahlung

Richtige Ernährung bei Krebs - Das können Sie selbst tun

Strahlentherapie natürlich unterstützen. Infos & Empfehlungen für Betroffene & Angehörige. Dr. Hüther informiert Sie über Nebenwirkungen und Therapieunterstützung bei Bestrahlung.

Die richtige Ernährung · Die 4-Schritte-Anleitung · Chemo-Nebenwirkungen

---

☐3

Krebsinformationsdienst
https://www.krebsinformationsdienst.de › iblatt PDF

Informationsblatt: Ernährung bei Krebs

Page 1. **ERNÄHRUNG BEI KREBS**: Was ist wichtig? y Eine individuell angepasste Ernährung kann die Ver- träglichkeit einer Krebsbehandlung und damit die Le-.

☐4

Gesponsert

Stärker gegen Krebs
https://www.staerkergegenkrebs.de

Ernährungsberater finden - in Ihrer Nähe & vergleichen

Erfahren Sie mehr zu dem Einfluss der **Ernährung** auf Ihre **Krebserkrankung**. Finden Sie Ihren Ernährungsberater vor Ort. Inklusive Informationen & Kontaktmöglichkeit. Therapien.

Was zahlen Krankenkassen · Unterstützung bei Krebs · Nebenwirkungen Lindern

---

☐5 Ich weiß es nicht

Sie suchen im Internet nach Informationen zu „Linderungsmöglichkeiten von krebsassoziierter Fatigue. Fatigue ist ein Zustand außerordentlicher Müdigkeit und mangelnder Energiereserven, der selbst nach angemessenen Ruhepausen nicht verschwindet. Welche der Links beinhaltet mit hoher Wahrscheinlichkeit die Informationen, zu dem Thema?

☐1

gesundheitsforschung-bmbf.de
https://www.gesundheitsforschung-bmbf.de › erschopf...

Erschöpfungssyndrom bei Krebskranken

Fatigue heißt diese besondere Form der Erschöpfung bei **Krebs**. Sie belastet viele Patienten auch noch Jahre nach Abschluss einer Tumortherapie. Wissenschaftler ...

☐2

Deutsche Krebsgesellschaft
https://www.krebsgesellschaft.de › fatigue-bei-krebs

Tumor-assoziierte Fatigue

Tritt eine anhaltende Erschöpfung im Zusammenhang mit einer **Krebserkrankung** auf, wird sie als Tumor-assoziierte **Fatigue** bezeichnet. Typisch für **Fatigue** sind ...

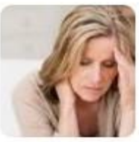

☐3

Deutsche Krebshilfe
https://www.krebshilfe.de › ... › Mit Krebs leben

Fatigue (Chronische Müdigkeit bei Krebs)

Fatigue ist eine langanhaltende, chronische Müdigkeit, die während und zum Teil noch längere Zeit nach der Behandlung einer **Krebserkrankung** besteht und auch ...

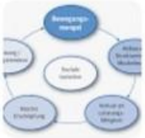

☐4

Krebsinformationsdienst
https://www.krebsinformationsdienst.de · vor 3 Jahren

Fatigue bei Krebs: Was hilft gegen extreme Müdigkeit?

Entspannungsübungen: Yoga, Massagen oder progressive Muskelentspannung können eine **Fatigue lindern**. Betroffene sollten überlegen, von welchem ...

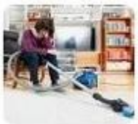

☐5 Ich weiß es nicht

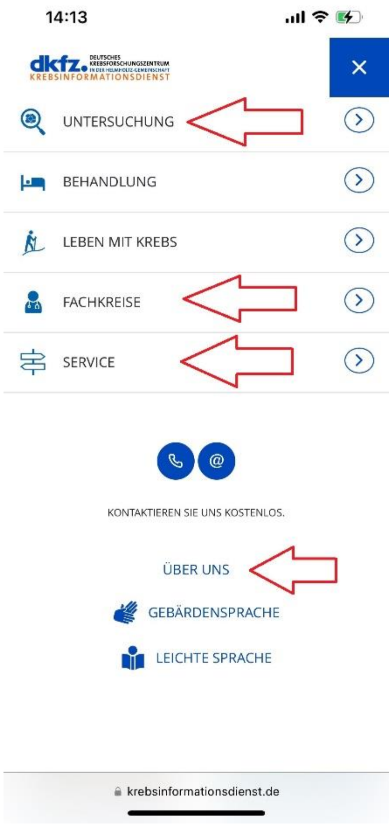

Stellen Sie sich vor Sie sind auf der Website des Krebsinformationsdienst und möchten wissen, was genau der Krebsinformationsdienst macht und wer dahinter steht. Auf welche Schaltfläche klicken Sie?

☐1 UNTERSUCHUNG

☐2 FACHKREISE

☐3 ÜBER UNS

☐4 SERVICE

☐5 Ich weiß es nicht

frontiers

Stellen Sie sich vor, dass sie als Krebspatient*in an chronischer Müdigkeit leiden. Ihr Partner vermutet, dass Sie an krebsassoziierter Fatigue leiden. Fatigue ist ein Zustand außerordentlicher Müdigkeit und mangelnder Energiereserven, der selbst nach angemessenen Ruhepausen nicht verschwindet. Sie möchten die genauen Symptome von krebsassoziierter Fatigue herausfinden. Eine Google-Suche führt sie zu den unten stehenden Ergebnissen. Welches dieser Ergebnisse würde Ihnen am ehesten eine korrekte und zuverlässige Antwort geben?

☐ 1

DocCheck Flexikon
https://flexikon.doccheck.com › Fatigue

Fatigue

Fatigue ist ein Syndrom (eine Ansammlung unterschiedlicher Symptome), das als Begleiterscheinung verschiedener chronischer Krankheiten auftritt, ...

☐ 2

BILD.de
https://www.bild.de › Leben & Wissen › Medizin

Müdigkeit, Gewichtsverlust: Diese Krebs-Symptome ...

12.06.2012 — Sind Sie immer **müde**? Haben Sie abgenommen oder gar unerklärliche Schmerzen? Dann sollten Sie schleunigst zum Arzt gehen. Es könnte **Krebs** ...

☐ 3

Deutsche Krebsgesellschaft
https://www.krebsgesellschaft.de › onko-internetportal

Fatigue bei Krebs - Überblick

Übermäßige Schonung, so der aktuelle wissenschaftliche Stand, hat keinen positiven Effekt auf die **Fatigue-Symptome**. ... **krebsassoziierte-fatigue**-chronische ...

☐ 4

Rheuma-Liga
https://www.rheuma-liga.de › rheuma › krankheitsbilder

Fatigue-Syndrom: Kampf gegen Müdigkeit und Erschöpfung

Viele rheumatische Erkrankungen gehen mit **Fatigue** einher. Die Ursachen für die andauernde Erschöpfung sind noch nicht geklärt.

☐ 5 Ich weiß es nicht

Sie sind in einem Forum für Krebspatienten und lesen folgende 3 Beiträge. Welcher dieser fiktiven Beiträge verstößt gegen die Privatsphäre und verletzt den Datenschutz?

☐1 Hallo an Alle,
Meine Mutter heißt Marie Schäfer und ist 56 Jahre alt. Vor einem halben Jahr hat sie die Diagnose Brustkrebs bekommen und wird zurzeit mit Chemo therapiert. Sie ist bei Herrn Dr. Schneider im Klinikum Bremen Mitte in Behandlung. Hat jemand dort schon Erfahrung gesammelt?

☐2 Moin moin,
Hat jemand hier Erfahrungen mit alternativen Heilpraktiken in Bezug auf Darmkrebs gemacht?
LG Peter

☐3 Liebe Community,
ich hoffe, es geht euch allen so gut wie möglich. Ich wollte mich kurz vorstellen und meinen Weg teilen, um Unterstützung zu finden und vielleicht auch anderen Mut zu machen.
Vor einigen Wochen erhielt ich die Diagnose Krebs. Ich bin entschlossen, diesen Kampf anzunehmen und positiv voranzuschreiten. Dabei würde ich gerne von euren Erfahrungen hören, Tipps zur Bewältigung von Nebenwirkungen erhalten und auch einfach nur ein offenes Ohr finden.
Vielen Dank für eure Zeit und Unterstützung. Gemeinsam sind wir stärker!
Liebe Grüße, Sandra

☐4 Ich weiß es nicht

Version for MacOS users:

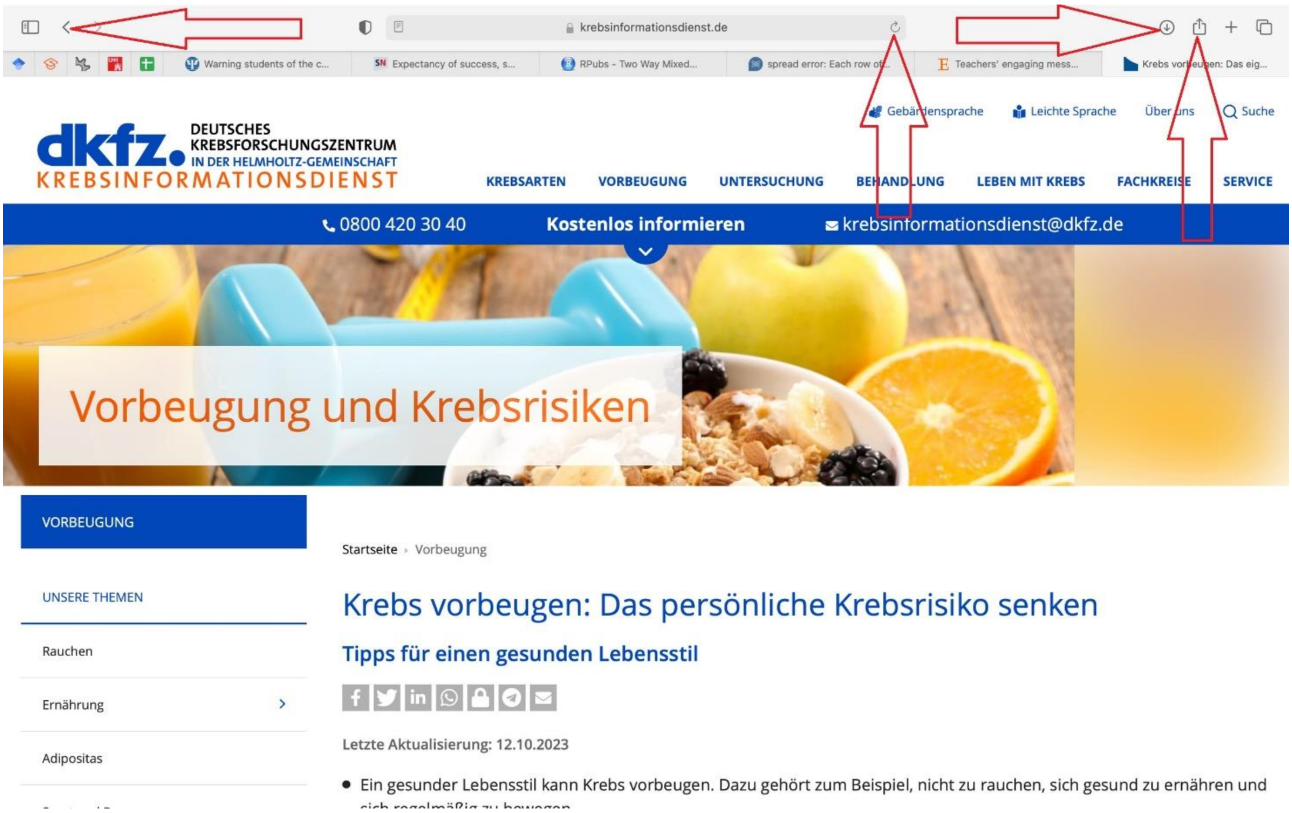

Sie sind über die Startseite des Krebsinformationsdienst auf die folgende Seite gelangt und möchten jetzt wieder zurück auf die Startseite. Wie kommen Sie zurück auf die Startseite?

☐ 1 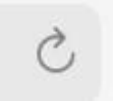

☐ 2 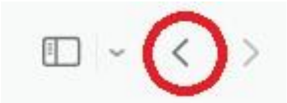

☐ 3 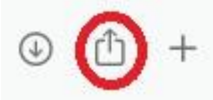

☐ 4 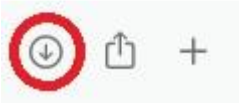

☐ 5 Ich weiß es nicht

Ihr Computer schreibt nur noch in Großbuchstaben. Was müssen Sie tun, um das wieder rückgängig zu machen?

☐1 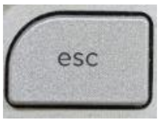

☐2 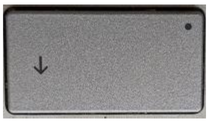

☐3 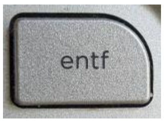

☐4 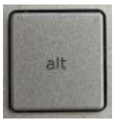

☐5 Ich weiß es nicht

Sie möchten sich von einer Internetseite Informationen kopieren. Wie machen Sie das?

☐1 Die gewünschten Informationen markieren und die Tasten „alt gr“ und „c“

☐2 Die gewünschten Informationen markieren und die Tasten „Strg“ und „c

☐3 Die gewünschten Informationen markieren und die Tasten „Strg“ und „k“

☐4 Die gewünschten Informationen markieren und die Tasten „alt gr“ und „k“

☐5 Ich weiß es nicht

Sie recherchieren bei Google Ernährungstipps zu ihrer Krebserkrankung. Bei welchen Seiten ist ein kommerzielles Interesse zu vermuten. Kreuzen sie an. (Es dürfen mehr als eine Antwortmöglichkeit ausgewählt werden).

☐1

Deutsche Krebsgesellschaft
https://www.krebsgesellschaft.de › basis-informationen...

Ernährung und Krebs

Erfahren Sie mehr über gesunde **Ernährung** zur Krebsprävention und welche Schadstoffe im Essen Sie meiden sollten um **Krebs** vorzubeugen.

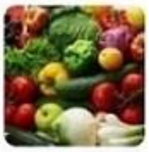

☐2

Gesponsert
Naturheilkompass
https://www.naturheilkompass.de › bestrahlung

Richtige Ernährung bei Krebs - Das können Sie selbst tun

Strahlentherapie natürlich unterstützen. Infos & Empfehlungen für Betroffene & Angehörige. Dr. Hüther informiert Sie über Nebenwirkungen und Therapieunterstützung bei Bestrahlung.
Die richtige Ernährung · Die 4-Schritte-Anleitung · Chemo-Nebenwirkungen

---

☐3

Krebsinformationsdienst
https://www.krebsinformationsdienst.de › iblatt PDF

Informationsblatt: Ernährung bei Krebs

Page 1. **ERNÄHRUNG BEI KREBS**: Was ist wichtig? y Eine individuell angepasste Ernährung kann die Ver- träglichkeit einer Krebsbehandlung und damit die Le-.

☐4

Gesponsert
Stärker gegen Krebs
https://www.staerkergegenkrebs.de

Ernährungsberater finden - in Ihrer Nähe & vergleichen

Erfahren Sie mehr zu dem Einfluss der **Ernährung** auf Ihre **Krebserkrankung**. Finden Sie Ihren Ernährungsberater vor Ort. Inklusive Informationen & Kontaktmöglichkeit. Therapien.
Was zahlen Krankenkassen · Unterstützung bei Krebs · Nebenwirkungen Lindern

---

☐5 Ich weiß es nicht

Sie suchen im Internet nach Informationen zu „Linderungsmöglichkeiten von krebsassoziierter Fatigue. Fatigue ist ein Zustand außerordentlicher Müdigkeit und mangelnder Energiereserven, der selbst nach angemessenen Ruhepausen nicht verschwindet. Welche der Links beinhaltet mit hoher Wahrscheinlichkeit die Informationen, zu dem Thema?

☐1

gesundheitsforschung-bmbf.de
https://www.gesundheitsforschung-bmbf.de › erschopf...

Erschöpfungssyndrom bei Krebskranken

Fatigue heißt diese besondere Form der Erschöpfung bei **Krebs**. Sie belastet viele Patienten auch noch Jahre nach Abschluss einer Tumortherapie. Wissenschaftler ...

☐2

Deutsche Krebsgesellschaft
https://www.krebsgesellschaft.de › fatigue-bei-krebs

Tumor-assoziierte Fatigue

Tritt eine anhaltende Erschöpfung im Zusammenhang mit einer **Krebserkrankung** auf, wird sie als Tumor-assoziierte **Fatigue** bezeichnet. Typisch für **Fatigue** sind ...

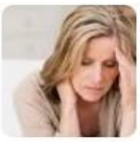

☐3

Deutsche Krebshilfe
https://www.krebshilfe.de › ... › Mit Krebs leben

Fatigue (Chronische Müdigkeit bei Krebs)

Fatigue ist eine langanhaltende, chronische Müdigkeit, die während und zum Teil noch längere Zeit nach der Behandlung einer **Krebserkrankung** besteht und auch ...

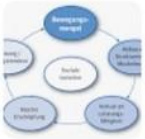

☐4

Krebsinformationsdienst
https://www.krebsinformationsdienst.de · vor 3 Jahren

Fatigue bei Krebs: Was hilft gegen extreme Müdigkeit?

Entspannungsübungen: Yoga, Massagen oder progressive Muskelentspannung können eine **Fatigue lindern**. Betroffene sollten überlegen, von welchem ...

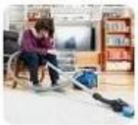

☐5 Ich weiß es nicht

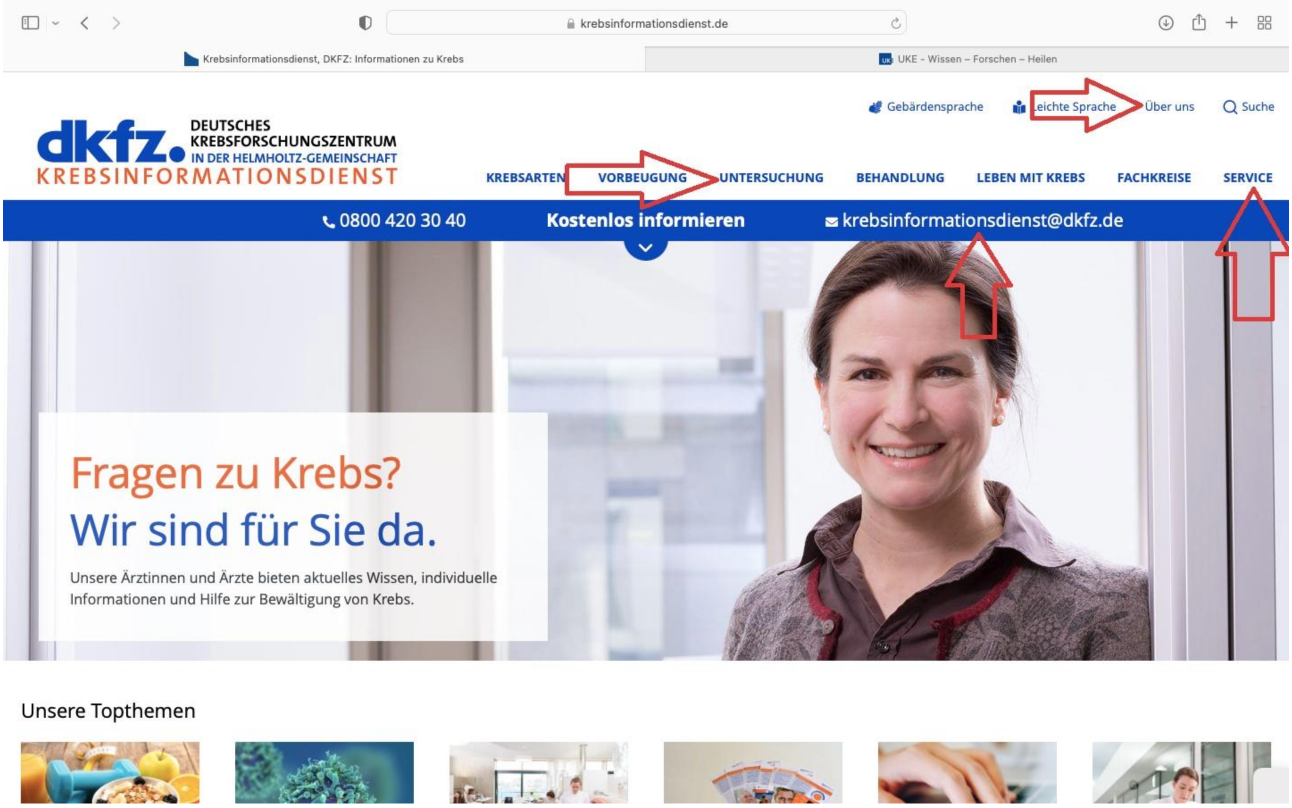

Stellen Sie sich vor Sie sind auf der Website des Krebsinformationsdienst und möchten wissen, was genau der Krebsinformationsdienst macht und wer dahinter steht. Auf welche Schaltfläche klicken Sie?

☐1 Über uns

☐2 UNTERSUCHUNG

☐3 SERVICE

☐4 krebsinformationsdienst@dkfz.de

☐5 Ich weiß es nicht

Stellen Sie sich vor, dass sie als Krebspatient*in an chronischer Müdigkeit leiden. Ihr Partner vermutet, dass Sie an krebsassoziierter Fatigue leiden. Fatigue ist ein Zustand außerordentlicher Müdigkeit und mangelnder Energiereserven, der selbst nach angemessenen Ruhepausen nicht verschwindet. Sie möchten die genauen Symptome von krebsassoziierter Fatigue herausfinden. Eine Google-Suche führt sie zu den unten stehenden Ergebnissen. Welches dieser Ergebnisse würde Ihnen am ehesten eine korrekte und zuverlässige Antwort geben?

☐1

DocCheck Flexikon
https://flexikon.doccheck.com › Fatigue

Fatigue

Fatigue ist ein Syndrom (eine Ansammlung unterschiedlicher Symptome), das als Begleiterscheinung verschiedener chronischer Krankheiten auftritt, ...

☐2

BILD.de
https://www.bild.de › Leben & Wissen › Medizin

Müdigkeit, Gewichtsverlust: Diese Krebs-Symptome ...

12.06.2012 — Sind Sie immer **müde**? Haben Sie abgenommen oder gar unerklärliche Schmerzen? Dann sollten Sie schleunigst zum Arzt gehen. Es könnte **Krebs** ...

☐3

Deutsche Krebsgesellschaft
https://www.krebsgesellschaft.de › onko-internetportal

Fatigue bei Krebs - Überblick

Übermäßige Schonung, so der aktuelle wissenschaftliche Stand, hat keinen positiven Effekt auf die **Fatigue-Symptome**. ... **krebsassoziierte-fatigue**-chronische ...

☐4

Rheuma-Liga
https://www.rheuma-liga.de › rheuma › krankheitsbilder

Fatigue-Syndrom: Kampf gegen Müdigkeit und Erschöpfung

Viele rheumatische Erkrankungen gehen mit **Fatigue** einher. Die Ursachen für die andauernde Erschöpfung sind noch nicht geklärt.

☐5 Ich weiß es nicht

Sie sind in einem Forum für Krebspatienten und lesen folgende 3 Beiträge. Welcher dieser fiktiven Beiträge verstößt gegen die Privatsphäre und verletzt den Datenschutz?

☐1 Hallo an Alle,
Meine Mutter heißt Marie Schäfer und ist 56 Jahre alt. Vor einem halben Jahr hat sie die Diagnose Brustkrebs bekommen und wird zurzeit mit Chemo therapiert. Sie ist bei Herrn Dr. Schneider im Klinikum Bremen Mitte in Behandlung. Hat jemand dort schon Erfahrung gesammelt?

☐2 Moin moin,
Hat jemand hier Erfahrungen mit alternativen Heilpraktiken in Bezug auf Darmkrebs gemacht?
LG Peter

☐3 Liebe Community,
ich hoffe, es geht euch allen so gut wie möglich. Ich wollte mich kurz vorstellen und meinen Weg teilen, um Unterstützung zu finden und vielleicht auch anderen Mut zu machen.
Vor einigen Wochen erhielt ich die Diagnose Krebs. Ich bin entschlossen, diesen Kampf anzunehmen und positiv voranzuschreiten. Dabei würde ich gerne von euren Erfahrungen hören, Tipps zur Bewältigung von Nebenwirkungen erhalten und auch einfach nur ein offenes Ohr finden.
Vielen Dank für eure Zeit und Unterstützung. Gemeinsam sind wir stärker!
Liebe Grüße, Sandra

☐4 Ich weiß es nicht
